# Hepatogenic Potential and Liver Regeneration Effect of Human Liver-derived Mesenchymal-Like Stem Cells

**DOI:** 10.3390/cells9061521

**Published:** 2020-06-22

**Authors:** Jooyoung Lee, Jiwan Choi, Seoon Kang, Jiye Kim, Ryunjin Lee, Seongjun So, Young-In Yoon, Varvara A. Kirchner, Gi-Won Song, Shin Hwang, Sung-Gyu Lee, Eunju Kang, Eunyoung Tak

**Affiliations:** 1 Department of Convergence Medicine, Asan Medical Institute of Convergence Science and Technology (AMIST), Asan Medical Center, University of Ulsan College of Medicine, Seoul 05505, Korea; leejeyre@naver.com (J.L.); jiwanc624@gmail.com (J.C.); tarxiens@gmail.com (S.K.); gilkja@naver.com (J.K.); soseongjun7@gmail.com (S.S.); 2 Biomedical Research Center, Asan Institute for Life Sciences, and Asan-Minnesota Institute for Innovating Transplantation (AMIT), Asan Medical Center, University of Ulsan College of Medicine, Seoul 05505, Korea; cordelia6070@gmail.com; 3 Stem Cell Center, Asan Institute for Life Sciences, and Asan-Minnesota Institute for Innovating Transplantation (AMIT), Asan Medical Center, University of Ulsan College of Medicine, Seoul 05505, Korea; 4 Division of Liver Transplantation and Hepatobiliary Surgery, Department of Surgery, and Asan-Minnesota Institute for Innovating Transplantation (AMIT), University of Ulsan College of Medicine, Seoul 05505, Korea; youngin83@naver.com (Y.-I.Y.); drsong71@amc.seoul.kr (G.-W.S.); shwang@amc.seoul.kr (S.H.); sglee2@amc.seoul.kr (S.-G.L.); 5 Division of Transplantation, Department of Surgery, and Asan-Minnesota Institute for Innovating Transplantation (AMIT), University of Minnesota, Minneapolis, MN 55455, USA; kirc0079@umn.edu

**Keywords:** human liver-derived stem cell, hepatic differentiation, hepatocyte-like cell, acute liver injury, regenerative medicine, cell transplantation

## Abstract

Human liver-derived stem cells (hLD-SCs) have been proposed as a possible resource for stem cell therapy in patients with irreversible liver diseases. However, it is not known whether liver resident hLD-SCs can differentiate toward a hepatic fate better than mesenchymal stem cells (MSCs) obtained from other origins. In this study, we compared the differentiation ability and regeneration potency of hLD-SCs with those of human umbilical cord matrix-derived stem cells (hUC-MSCs) by inducing hepatic differentiation. Undifferentiated hLD-SCs expressed relatively high levels of endoderm-related markers (*GATA4* and *FOXA1*). During directed hepatic differentiation supported by two small molecules (Fasudil and 5-azacytidine), hLD-SCs presented more advanced mitochondrial respiration compared to hUC-MSCs. Moreover, hLD-SCs featured higher numbers of hepatic progenitor cell markers on day 14 of differentiation (*CPM* and *CD133*) and matured into hepatocyte-like cells by day 7 through 21 with increased hepatocyte markers (*ALB*, *HNF4A,* and *AFP*). During in vivo cell transplantation, hLD-SCs migrated into the liver of ischemia-reperfusion injury-induced mice within 2 h and relieved liver injury. In the thioacetamide (TAA)-induced liver injury mouse model, transplanted hLD-SCs trafficked into the liver and spontaneously matured into hepatocyte-like cells within 14 days. These results collectively suggest that hLD-SCs hold greater hepatogenic potential, and hepatic differentiation-induced hLD-SCs may be a promising source of stem cells for liver regeneration.

## 1. Introduction

Liver replacement therapy or liver transplantation is considered a gold standard for patients with end-stage liver disease [1,2]. However, the shortage of available organs, high cost, risk of graft rejection, and the requirement of lifelong immunosuppression, which weakens the immune surveillance system and increases the incidence of tumor formation [3], makes it mandatory to seek alternate restorative approaches. Recent progress in stem cell technology has provided more expandable sources of liver cells for regenerative medicine and has promoted studies regarding their potential therapeutic application [4].

For cell therapy in liver injury, the hepatocyte itself has been primarily investigated. However, there are several limitations—hepatocytes rapidly lose certain functions when cultured in conventional environments and exhibit substantial loss after being frozen and thawed; moreover, there is restricted access to the human liver to obtain sufficient hepatocytes [5]. Alternatively, embryonic stem cells (ESCs) were considered at one point a possible source for hepatocyte-like cells because of their multipotency. Nevertheless, their use remains restricted in many countries owing to legal issues and ethical concerns [6]. Reprogrammed cells or induced pluripotent stem cells (iPSCs) are widely regarded as the alternative to ESCs; however, their drawbacks include high cost and safety concerns that constrain their wider use for further clinical applications [7].

Mesenchymal stromal/stem cells (MSCs) can be isolated from diverse tissues [8] and have stem cell markers [9], low immunogenicity [10,11], and differentiation potential [12]. Importantly, MSCs are essentially trophic and themselves can exert regenerative effects on damaged tissues, including the liver [13]. For example, previous studies have reported that systemic administration of MSCs resulted in their recruitment to the ischemic or injured liver area [14,15,16]. Moreover, MSCs are known to secrete liver regeneration-related growth factors and can support liver regeneration by relieving oxidative stress, suppressing apoptotic processes, increasing angiogenesis, and improving cell proliferation [17]. Based on these findings, MSCs obtained from various organs have recently been explored as a more acceptable source of hepatocyte-like cells, especially with their ability to differentiate towards hepatogenic lineages [18,19,20].

In the liver, hepatocytes are thought to originate from at least two different sources. One is the hepatic progenitor cells (also known as oval cells in rodents). These cells differentiate into hepatocytes and biliary epithelial cells and are activated when the liver is exposed to acute [21] or chronic injury [22]. Rodent oval cells and human hepatic progenitor cells express hematopoietic stem cell related markers [23,24], which suggests that hepatic progenitor cells might originate from hematopoietic stem cells. However, the origin of the hepatic progenitor cell is not clearly understood and is still under debate [22]. Hepatic progenitor cells are known to localize at the canal of Hering or intrahepatic bile ductules, the smallest branches of the intrahepatic biliary tree. They have been widely investigated for potential use in chronic and acute liver injuries. These days, these bipotent cells are considered an ideal source for liver regeneration therapy with high differentiation potency and expandability. Indeed, recent studies on liver regenerative therapy have used chemical approaches to de-differentiate adult liver cells into hepatic bipotent cells [25,26,27]. Another current trend for stem cell therapy is to use chemicals rather than viruses or gene-editing techniques to avoid unexpected adverse effects caused by modifying living organisms [28]. Collectively, it is obvious that hepatic progenitor cells are a promising source for liver regenerative medicine.

The other suggested source for hepatocytes is human liver-derived mesenchymal-like stem cells (hLD-SCs), which are different from hepatic progenitor cells and negative for the hematopoietic stem cell marker, CD34. The existence and basal characteristics of hLD-SCs were reported by Herrera et al. in 2006 [29] and Najimi et al. in 2007 [30]. Pan [31] and Lee [32] also reported similar hLD-SCs and investigated their characteristics. Cells with similar properties were observed in the rodent liver [33]. hLD-SCs were discussed in reviews by Wang et al. [34] and Kholodenko et al. [35], which summarized their MSC-like characteristics, including differentiation ability, immunosuppressive effect, and regenerative effect in damaged tissues. However, many questions remain surrounding further applications of hLD-SCs.

In the current study, we followed up on the changes in the intracellular dynamics during hepatic differentiation of hLD-SCs. Additionally, as emerging evidence on small molecules suggested the benefits of maintaining or controlling the fate of MSCs, we combined small molecules to induce stable hepatic differentiation of stem cells [36]. Small molecules can target specific signaling pathways to modulate the fate and function of stem cells [37]. In keeping with this, in this work, we used two different small molecules. The first is Fasudil, a RhoA/ROCK signaling inhibitor. This molecule is known to maintain confluent human MSCs by inhibiting cell apoptosis and increasing the efficiency of endoderm differentiation [38,39]. The other is 5-azacytidine, a DNA methyltransferase (DNMT) inhibitor. 5-azacytidine is known to increase the expression of hepatic-associated marker genes and enhances the hepatic function of differentiated hepatocyte-like cells [40]. We also measured mitochondrial function and metabolic status of hLD-SCs during hepatic differentiation. Spontaneous events of in vivo differentiation and maturation of hLD-SCs into functional hepatocytes during liver injury induced in mice at different time points of hepatic differentiation were also observed. Altogether, the present study highlights the hepatogenic potential of hLD-SCs along with the significant therapeutic potential of hepatic differentiation-induced hLD-SCs.

## 2. Materials and Methods

### 2.1. Isolation of hLD-SCs from Human Liver Tissues

We obtained human livers from donors who visited the Asan Medical Center (Seoul, Korea) for living donor liver transplantation and who agreed to provide a specimen for research. The clinical characteristics of the human liver donors are listed in Table 1. To isolate hLD-SCs, small pieces of liver tissue were transferred on ice, suspended in 5 mL of Dulbecco’s modified Eagle’s medium (DMEM)/F12 (1:1) with HEPES (Hyclone, GE Healthcare Life Sciences, Logan, UT, USA), and successively centrifuged at 500× *g* for 3 min. If necessary, specimens were transferred to a Petri dish and cut into small pieces under sterile conditions. The supernatant was removed, and 5 mL of 0.1% collagenase IV solution (Gibco, Life Technologies Corporation, Grand Island, NY, USA) was added. The tissue was incubated with the collagenase solution for 30 min and dissociated mechanically by gentle pipetting. After digestion, 5 mL of DMEM/F12 supplemented with 10% fetal bovine serum (FBS; Gibco) was added, and the suspension was strained with a 70-μm cell strainer (Becton Dickinson and Company, Franklin Lakes, NJ, USA). The filtered cells were transferred to 15 mL centrifuge tubes, washed by adding 10 mL medium and pelleted by centrifugation at 400× *g* for 5 min 3 times. The cells obtained were suspended in the plating medium supplemented with basic fibroblast growth factor (bFGF; Peprotech, Rocky Hill, NJ, USA) by gentle pipetting, counted, and seeded on a gelatin-coated culture plate. During maintaining and passaging hLD-SCs, no selection process was involved. This work was approved by the Institutional Review Board of Asan Medical Center (authorization no. 2018-1386). All volunteers provided written informed consent. The research was conducted in accordance with the Helsinki Declaration.

### 2.2. Preparation of Human Umbilical Cord Matrix-Derived Stem Cells (hUC-MSC) and Human Bone Marrow Stem Cells (hBM-MSC)

The hUC-MSCs and hBM-MSC were provided by the Stem Cell Center (Asan Institute for Life Sciences, Asan Medical Center, Seoul, Korea) [41,42]. Briefly, the human umbilical cord, not including vessels or amnions, was chopped and digested in minimal essential medium (MEM; Gibco) with 0.1% collagenase A (Roche, Indianapolis, IN, USA) for 3 h at 37 °C with shaking. Then, the cells were filtered using a 70 μm strainer and pelleted by centrifuging at 200× *g* for 10 min. The collected cells were seeded in DMEM (Gibco) supplemented with 10% FBS, 100 U/mL penicillin, and 100 μg/mL streptomycin, and maintained at 37 °C in a 5% CO_2_ humidified incubator.

For hBM-MSCs, the bone marrow aspirates were obtained from the human iliac crest, diluted in a 1:1 ratio with Dulbecco’s phosphate-buffered saline (DPBS; Gibco), and layered on Ficoll-Paque PLUS (density 1.077 g/mL; GE Healthcare, Piscataway, NJ, USA). The mononuclear cells were obtained by density gradient centrifugation at 400× *g* for 30 min at room temperature and cultured under the same conditions as those used for hUC-MSCs [43].

For all stem cells, the growth medium was changed every 3 days until cells grew 80% confluent, at which point the non-adherent cells were removed. The stem cell monolayer was detached using 0.25% trypsin-EDTA solution (Gibco). All further analyses involving hUC-MSCs, hBM-MSCs, and hLD-SCs were performed at passages 4–6 and pooled at passages 4 and 5 for further investigation.

### 2.3. Flow Cytometric Analysis of Human Stem Cells

Cell surface proteins were measured by flow cytometry using previously described methods [43]. The hBM-MSCs, hUC-MSCs, and 3 different hLD-SCs were sequentially incubated with primary antibodies in refrigerated blocking buffer for 1 h and secondary immunofluorescent antibodies. Antibodies against PE-labeled CD34 (BD Biosciences, Franklin Lakes, NJ, USA), FITC-labeled CD90 (Abcam, Cambridge, MA, USA), and CD105 (Abcam) were used while the CD34-positive cells were also parallelly examined (Supplementary Figure S1A). Cells incubated with a blocking buffer without primary antibodies were used as a negative control. A total of 10,000 events were evaluated with the BD FACScanto II (Becton Dickinson and Company) and analyzed using the FlowJo software (ver 10.6.1; Treestar, Ashland, OR, USA).

### 2.4. Total mRNA Extraction and qRT-PCR Analysis

Total RNA of all stem cells, stage-wise hepatic differentiation-induced cells, and isolated human primary hepatocytes (PHH) were obtained using an RNeasy Mini Kit (Qiagen, Valencia, CA, USA) following the manufacturers’ instructions. For liver tissues, roughly 40–50 mg of tissues were prepared, and QIAzol lysis solution was used to extract mRNA from samples (Qiagen). The complementary DNA (cDNA) was synthesized using a ReverTra Ace qPCR RT Master Mix (Toyobo, Osaka, Japan), and qRT-PCR was performed with 5× HOT FIREPol EvaGreen qPCR Supermix (Solis BioDyne, Tartu, Estonia) using a CFX Connect Real-Time PCR Detection System (Bio-Rad Laboratories, Hercules, CA, USA). The samples were denatured at 95 °C for 15 min followed by 40 cycles of denaturation at 95 °C for 15 s, annealing at 60 °C for 20 s, and elongation at 72 °C for 20 s. The cycle threshold (CT) values and relative normalized expression were automatically determined using Bio-Rad CFX Maestro software (CFX Maestro™ version 1.1; Bio-Rad Laboratories). The specific amplification was measured with melt-curve analysis. The relative expression values were calculated based on the delta-delta Ct method and normalized to *GAPDH* expression. The primer sequences are specified in Table 2 and Supplementary Table S1.

### 2.5. Mixed Lymphocyte Reaction (MLR) Assay

For one-way MLR assays, peripheral blood mononuclear cells (PBMCs) were isolated from 2 different healthy volunteers (Blood donor 1: 36-year-old female, Asian; Blood donor 2: 29-year-old female, Asian) by density gradient centrifugation using Ficoll-Paque PLUS (GE Healthcare, Piscataway, NJ, USA). One lot of PBMCs were treated with 10 μg/mL mitomycin C (Sigma Aldrich, St. Louis, MO, USA) solution to prepare stimulated cells. After 1 h, the cells were washed with RPMI 1640 (Hyclone) supplemented with 10% FBS at least 3 times. Autologous or allogeneic MLR assays were performed by co-culturing PBMCs obtained from identical individuals or PBMCs from different individuals in 96-well flat-bottom plates (Corning Incorporated – Life Sciences, Durham, NC, USA) (1.0 × 10^5^ cells/well), respectively. The cells were incubated at 37 °C with 5% CO_2_ for 9 days. To measure the effect of stem cells on immune cell proliferation, hBM-MSCs, hUC-MSCs, and 3 hLD-SCs were passaged, counted, seeded, and co-cultured with PBMCs (1.0 × 10^5^ cells/well) for 96 h. To measure the proliferation index, viable cells were counted using the Viability Assay Kit (MediFab, Seoul, South Korea) according to the manufacturer’s instructions. Briefly, cell viability solution was added to each well before adding stem cells and 96 h after co-culturing with stem cells. After a 2 h incubation, the optical density was measured at a wavelength of 450 nm. The proliferation index was calculated by dividing the mean optical density values of cultured cells after 96 h with the mean optical density value of cells before the addition of stem cells (n = 4).

### 2.6. Immunofluorescence Assay

Cells were fixed with 4% formaldehyde after 0, 7, 14, and 21 days of differentiation. The fixed cells were washed, permeabilized, blocked, and incubated with albumin (1:100; Santacruz Biotechnology, Dallas, TX, USA) antibodies overnight at 4 °C. A goat anti-mouse IgG FITC-conjugated secondary antibody (1:100; Santacruz Biotechnology) was used to detect the signals. The nucleus was stained with 4′,6-diamidino-2-phenylindole (DAPI) mounting solution (Abcam). The fluorescence signals were detected with a fluoroscopic microscope (AxioObserver Z1; Carl Zeiss, Oberkochen, Germany).

### 2.7. In Vitro Hepatic Differentiation of Stem Cells

The 2-step differentiation protocol for hepatocyte differentiation was simply modified from previously reported hepatogenic differentiation protocols for MSCs [19]. Briefly, each stem cell type was seeded in a 25 cm^2^ T flask (Thermo Fisher Scientific, Waltham, MA, USA) and maintained in DMEM/F12 (1:1; Hyclone) supplemented with 10 ng/mL bFGF (Peprotech), 1% non-essential amino acids (NEAA; Gibco), 100 U/mL penicillin, 100 μg/mL streptomycin, and 10% FBS (Gibco) at 37 °C with 5% CO_2_ in a humidified incubator. After 2 days, the cells were incubated with Step-1 medium consisting of Iscove’s modified Dulbecco’s medium (IMDM; Gibco) supplemented with 20 ng/mL HGF (Peprotech), 10 ng/mL bFGF, 0.61 mg/mL nicotinamide (Sigma Aldrich) for 7 days. Two small molecules, 2 μM of 5-azacytidine (Sigma Aldrich), and 10 μM of Fasudil-HCl (AdooQ bioscience, Irvine, CA, USA) were added during the Step-1 differentiation. For hepatic maturation, the medium was changed to Step-2 medium consisting of IMDM supplemented with 20 ng/mL oncostatin M (R&D systems, Minneapolis, MN, USA), 1 μM dexamethasone (Sigma Aldrich), and 50 ng/mL ITS (insulin-transferrin-selenium; Gibco) for up to 21 days. All media were changed every other day, and morphological changes were microscopically monitored (Olympus IX73; Olympus, Tokyo, Japan).

### 2.8. Mitochondrial Function Measurement (Seahorse Assay)

Mitochondrial respiratory function was measured using an XF Cell MitoStress test kit in an XF24 extracellular flux analyzer (Agilent Technologies, Santa Clara, CA, USA). All day 0 and day 7 cells were seeded on gelatin-coated XFe24 cell culture plates (Agilent Technologies) before mitochondrial oxygen consumption rate (OCR) measurement. As it was difficult to detach day 14 cells from the originally cultured plate, all day 14 hepatocyte-like cells were differentiated on gelatin-coated XFe24 cell culture plates from the first day. Mitochondrial OCR was measured by the serial addition of oligomycin (1.5 μM), carbonyl cyanide 4-(trifluoromethoxy) phenylhydrazone (FCCP; 1 μM), and antimycin A (0.5 μM) with rotenone (0.5 μM). ATP production, maximal respiration, reverse capacity, and non-mitochondrial oxygen utilization were calculated by previously described methods [44]. The values were normalized to baseline oxygen consumption and the amount of cellular DNA.

### 2.9. Liver Injury Models with In Vivo Stem Cell Treatments

For all in vivo experiments, hLD-SC 1 and hLD-SC 2 were used as representative hLD-SCs. To measure short-term trafficking of stem cells, a liver ischemia-reperfusion injury (LIRI) mouse model was utilized. Male 8-week-old C57BL/6J mice were purchased from Joongah Bio (Gyeonggi-do, Korea). To induce partial hepatic ischemia, an 11 mm long micro-clamp (cat no. 00398-02; Fine Science Tools, Heidelberg, Germany) was used to block the portal vein for 45 min and then released. After removal of the clamp, mice were treated with 1.0 × 10^6^ cells via tail-vein injection and then reperfused for 6 h.

To measure the spontaneous differentiation of stem cells in vivo, 8-week-old immune-deficient Rag2^-/-^Il2rg^-/-^ (NRG) mice were purchased from Joongah Bio. To induce acute liver failure, mice were injected intraperitoneally with the hepatotoxic chemical, thioacetamide (TAA; 80 mg/kg) twice over the course of 5 days. On the 5th day, mice were treated with hUC-MSCs as well as hLD-SC 1 or hLD-SC 2 cells after 0, 7, and 14 days of hepatic differentiation via the intrasplenic route (1.0 × 10^6^ cells per mouse). All mice were allowed to recover for 14 days after cell transplantation.

All animal experiments were performed in accordance with relevant guidelines and regulations of the Institute of Laboratory Animal Resources (ILAR). The study protocol was reviewed and approved by the Institutional Animal Care and Use Committee (IACUC), Asan Institute for Life Sciences, Asan Medical Center (Authorization no. 2018-12-167).

### 2.10. Cell Labeling with DiR and In Vivo Fluorescence Imaging

A total of 1.0 × 10^6^ cells was labeled with XenoLight DiR (Perkin Elmer, Waltham, MA, USA). The staining procedure was performed according to the manufacturer’s instructions. Briefly, cells were treated with DiR for 30 min at 37 °C, centrifuged for 3 min at 400 × *g* at room temperature, and washed twice with PBS. In all cases, DiR-labeled cells were suspended in saline and then intravenously injected through the tail vein within 2 h of labeling. Before taking the fluorescence images, the hair of C57BL/6 mice was removed to avoid any interference in detecting the fluorescence signal. Fluorescence images were obtained at 1, 2, 4, and 6 h after injection using the Xenogen IVIS Spectrum system (Perkin Elmer). All images were acquired with excitation at 748 nm and emission at 780 nm.

### 2.11. Serum Measurement Assays

Whole-blood samples were obtained from the inferior vena cava of the mice and coagulated in serum separator tubes (SSTs; Becton Dickinson and Company). The SST tubes were incubated for 30 min at room temperature (22 ± 2 °C) and centrifuged at 2500× *g* for 20 min at 4 °C. The supernatants were collected and stored as serum samples. Serum alanine aminotransferase (ALT) and aspartate aminotransferase (AST) levels were analyzed by a Hitachi 7180 autoanalyzer (Tokyo, Japan).

### 2.12. Detection of Human DNA in Mouse Livers

To measure the remaining human cells in mice treated with human stem cells, human-specific mitochondrial DNA (mtDNA) and human-specific genomic DNA (gDNA) was detected using PCR. Total DNA was extracted from formalin-fixed, paraffin-embedded tissue samples. The fixed samples were cut using a microtome, deparaffinized with xylene, washed with absolute ethanol, and then lysed using FavorPrep™ Tissue Genomic DNA Extraction Mini Kit (Cat no. FATGK001-2; Favorgen, Ping-Tung, Taiwan). For positive human samples, the commercially available normal liver cell line, HepaRG™ (1 × 10^5^ cells; Cat no. HPRGC10; Gibco) was utilized, and total DNA was extracted according to the same methods. Mouse genomic *GAPDH* was used as an internal control. The primer sequences are listed in Table 2.

### 2.13. Histological Analysis and Immunohistochemistry

Liver tissues were resected, fixed in 4% formalin, and embedded in paraffin. The paraffin blocks were sectioned with a microtome to produce 4 μm-thick sections. To assess the severity of liver injury, the H&E-stained slides were scored using Suzuki’s method with historical criteria for the assessment of liver damage after severe ischemia and reperfusion injury based on the presence and severity of sinusoidal congestion, cytoplasmic vacuolization, and necrosis of parenchymal cells by 3 pathologists (Table 3; score range, 0 – 4) [45].

For immunohistochemistry (IHC), the sections were deparaffinized in xylene, rehydrated using an alcohol gradient, boiled in 10 mM citrate buffer (pH 6.0) for 20 min to retrieve antigens, and then treated with 0.3% hydrogen peroxide in methanol to remove endogenous peroxidase. The Vectastain^®^ Elite^®^ ABC HRP Kit (Cat no. PK-7200; Vector Laboratories, Burlingame, CA, USA) was used in accordance with the manufacturer’s instructions. The STEM121™ (1:1,000; Cat no. Y40410; Takara Bio Inc., Kusatsu, Shiga, Japan) and albumin antibody (1:200; Cat no. sc-271605, Santacruz Biotechnology) was diluted in Dako REAL antibody diluent (Cat no. S202230-2; Agilent Technologies) and incubated overnight at 4 °C. To visualize the horseradish peroxidase (HRP) signals, DAB+ (Cat no. K346811-2; Agilent Technologies) was used according to the manufacturer’s instructions. The sections were counterstained with hematoxylin QS (Vector Laboratories) and mounted with VectaMount (Vector Laboratories). Histological images were obtained with light microscopy using a camera (Olympus DP27; Olympus, Melville, NY, USA). The areas of positive staining were measured using Image J software (NIH) in images with 100× magnification.

### 2.14. Statistical Analysis

All statistical analyses were performed using GraphPad Prism 6.0 software (GraphPad Software, San Diego, CA, USA). Histological scores were presented as the median and range. All other data were presented as the mean ± standard deviation (SD). Statistical analyses with 3 or more comparison sets were performed using 1-way or 2-way analysis of variance (ANOVA) followed by Bonferroni’s multiple comparison tests, and comparison with two groups was carried out with Student’s t-test. *P* < 0.05 was considered statistically significant.

## 3. Results

### 3.1. Characterization of hLD-SCs

**Figure 1:**
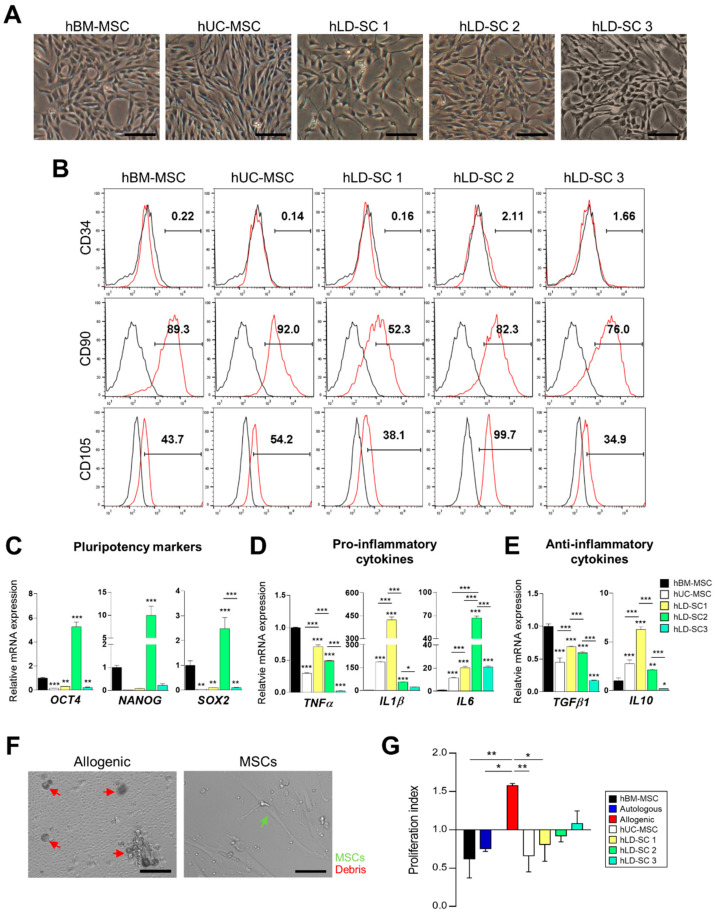
Comparison of the characteristics between human liver-derived stem cells (hLD-SCs) and mesenchymal stromal/stem cells (MSCs) from other origins. (**A**) Microscopy imaging of human umbilical cord matrix derived stem cell (hUC-MSCs), human bone marrow stem cell (hBM-MSCs), and human liver-derived stem cells (hLD-SCs) isolated from three different individuals. Scale bar, 200 μm. (**B**) Flow cytometry measurement of hUC-MSCs, hBM-MSCs, and three hLD-SCs. CD34 was labeled as a hematopoietic stem cell marker. CD90 and CD105 were labeled as mesenchymal stem cell markers. (**C**–**E**) Quantitative real-time PCR (qRT-PCR) analysis of (**C**) pluripotency markers (*OCT4*, *NANOG*, and *SOX2*), (**D**) pro-inflammatory cytokines (*TNFα*, *IL1β,* and *IL6*), and (**E**) anti-inflammatory cytokines (*TGFβ1* and *IL10*). Gene expression levels were normalized to *GAPDH* expression and compared to the expression levels of hBM-MSCs. Data are expressed as mean ± SD. n = 4. (**F**) Microscopy measurement of the interaction process between allogeneic peripheral blood mononuclear cells (PBMCs) isolated from two different volunteers without or with mesenchymal stromal/stem cells (MSCs). Scale bar, 100 μm. (**G**) Comparison of immune cell proliferation during MLR assay. Autologous, allogenic, and allogenic reactions after treatment with hBM-MSCs, hUC-MSCs or three hLD-SCs, respectively, were compared. The proliferation index was calculated by dividing absorbance of 96 h co-cultured cells at a wavelength of 450 nm to that of cells before treatment. *P*-values < 0.05 were considered significant. n = 4, *, *p* < 0.05; **, *p* < 0.01; ***, *p* < 0.001.

In terms of morphology, cultured hLD-SCs exhibited a stretched spindle shape with ovoid nuclei (Figure 1A). We observed morphological similarities between hBM-MSCs and hUC-MSCs. Flow cytometry showed that not only hUC-MSCs but also hLD-SCs expressed the MSC surface markers, CD90 and CD105, while all hUC-MSCs and hLD-SCs were negative for the hematopoietic stem cell marker, CD34 (Figure 1B; data from CD34-positive cells are available in Supplementary Figure S1A).

The hLD-SCs also expressed representative stem cell markers, namely octamer-binding transcription factor 4 (*OCT4*), *NANOG,* and sex-determining region Y - box 2 (*SOX2*) (Figure 1C). Compared to hBM-MSC, the expression levels of *OCT4*, *NANOG*, and *SOX2* were relatively low in hUC-MSCs as well as hLD-SC 1, and hLD-SC 3 cells whereas hLD-SC 2 cells were relatively highly expressed.

MSCs are known to be involved in both direct cell-to-cell contacts and indirect cell-to-cell communication by secreting cytokines, though the exact effects are not entirely known [46], thus we compared pro- and anti-inflammatory cytokines in those cells. Among pro-inflammatory cytokines, tumor necrosis factor *α* (*TNFα*) levels were significantly low in hUC-MSCs and all hLD-SCs compared to hBM-MSCs. Among hUC-MSCs and hLD-SCs, hLD-SC 3 cells expressed the lowest levels of *TNFα* and *IL1β*. However, *IL1β* was relatively high in hUC-MSCs and three types of hLD-SCs. All hUC-MSCs and hLD-SCs expressed higher levels of interleukin 6 (*IL6*) compared to hBM-MSCs whereas hLD-SC 2 cells expressed the highest levels (Figure 1D). The anti-inflammatory cytokine, transforming growth factor β1 (*TGFβ1*), was consistently low in hUC-MSCs and hLD-SCs compared to hBM-MSCs, while another anti-inflammatory cytokine, interleukin 10 (*IL10*), was relatively highly expressed in hUC-MSCs and two hLD-SCs with the highest expression in hLD-SC 1 cells. The expression of *IL10* in hLD-SC 3 cells was the lowest (Figure 1E).

The reasons for inconsistencies in cytokine expression are not clear, but may be because of the diversity of liver donors causing the differences. It is known that cytokine expression varies according to age, sex, and disease experiences [47]. Among three hLD-SCs, hLD-SC 1 were procured from a, 21-year-old male and expressed relatively high amounts of *TNFα*, *IL1β*, and *IL10*. The donor for hLD-SC 2 was a 38-year-old female and among the three liver donors, the triglyceride (TAG) contents in the 38-year-old donor’s serum was relatively high (157 mg/dl), though it did not exceed the normal range. hLD-SC 2 cells expressed the highest levels of pluripotency markers and *IL6*. hLD-SC 3 cells were isolated from a 27-year-old female with elevated serum total cholesterol (235 mg/dl) and high-density lipoprotein (HDL)-cholesterol (90 mg/dl). hLD-SC 3 cells expressed relatively low amounts of *TNFα*, *IL1β*, *TGFβ1,* and *IL10*. Although there were not enough cases to generalize the relationship between liver donors and obtained hLD-SCs, based on our evidence, the health status of the donor seems to be associated with the obtained hLD-SCs.

As MSCs suppress immune cell proliferation [48], we tested whether hLD-SCs might have similar immune regulation abilities. To induce immune cell proliferation, PBMCs obtained from two allogenic individuals were co-cultured. The allogeneic reactions and suppression of immune cell proliferation after treatment with each type of stem cell was observed under an inverted microscope (Figure 1F). Compared to the autologous immune cell proliferation rates, allogenic PBMCs showed significant (*p* < 0.001) proliferation. This proliferation was significantly (*p* < 0.001) decreased after treatment with hBM-MSCs, hUC-MSCs, or all three hLD-SCs (Figure 1G). Such results suggest an immunosuppressive effect of hLD-SCs, which is an important physiological function of MSCs.

### 3.2. The Modified Two-Step Hepatic Differentiation Protocol Produced Hepatocyte-Like Cells from hLD-SCs

**Figure 2:**
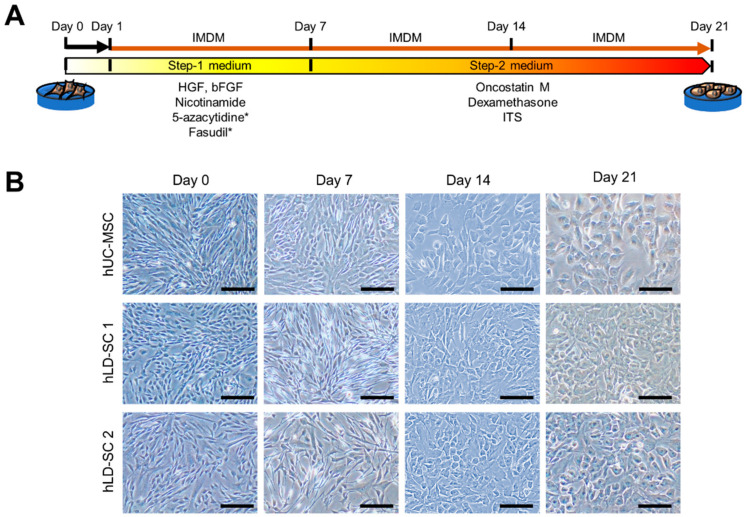
Modified two-step hepatic differentiation for stem cells changed hLD-SCs into those with a hepatocyte-like morphology. (**A**) Schematic summary of the methods for inducing two-step directed differentiation of stem cells into hepatocyte-like cells. *, small molecules. (**B**) Microscopic measurement during two-step hepatic differentiation. The hUC-MSCs and two hLD-SCs were measured. Cells on days 0, 7, 14, and 21 of hepatic differentiation are displayed. Scale bar, 200 μm.

A previous study has reported a two-step method to differentiate MSCs into hepatocyte-like cells with chemical compounds, including HGF, bFGF, and nicotinamide [19]. In the current study, we tested several mixtures of different chemicals and modified the previous two-step hepatocyte differentiation protocol by adding Fasudil, a RhoA/ROCK signaling inhibitor, and 5-azacytidine, a DNMT inhibitor to increase differentiation ability (Figure 2A). To measure the suitability of our differentiation protocols, the morphologic changes of cells were tracked. During Step-1 differentiation, morphologic changes were not apparent. However, 7 days after changing to the Step-2 cocktail, the cells started to become more rounded. Following the maintenance of cells in Step-2 medium for 7 more days, the cells exhibited robust changes into hepatocyte-like morphology (Figure 2B).

### 3.3. Comparison of Gene Expression Patterns during Hepatic Lineage Development

**Figure 3:**
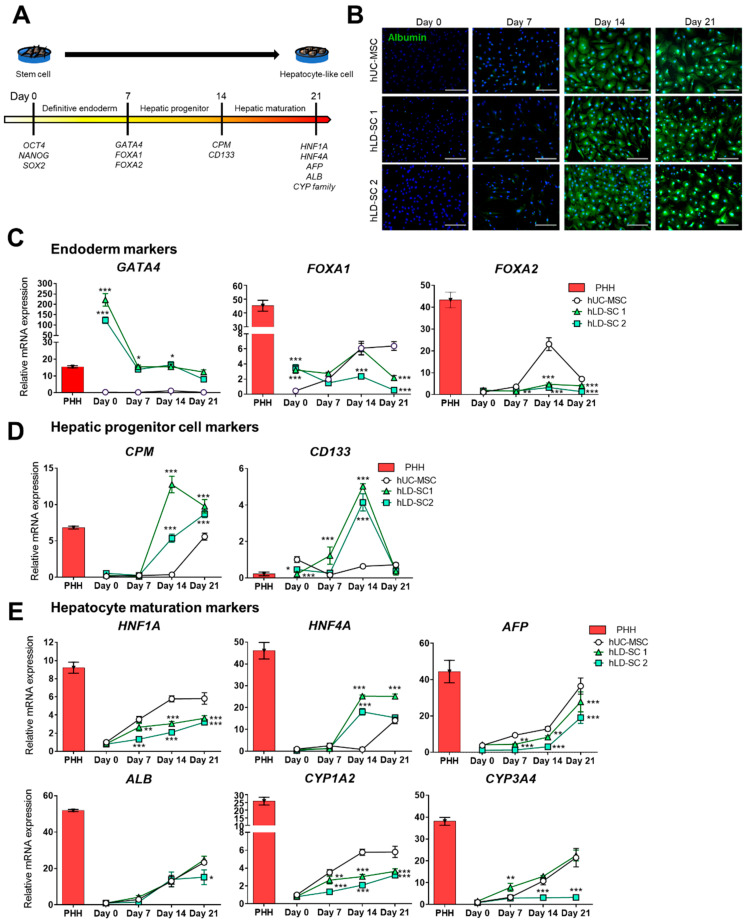
Expression of genes related to the differentiation of stem cells towards hepatic fate. (**A**) Schematic diagram of the differentiation of stem cells towards hepatic fate. The prominent genes during differentiation are described. (**B**) Albumin immunofluorescence staining of hUC-MSCs and two hLD-SCs on days 0, 7, 14, and 21 of differentiation. Blue, DAPI; Green, albumin. Scale bar, 200 μm. qRT-PCR measurement of (**C**) endodermal markers (*GATA4*, *FOXA1*, and *FOXA2*), (**D**) hepatic progenitor markers (*CPM* and *CD133*), and (**E**) hepatocyte maturation markers (*HNF1A*, *HNF4A*, *AFP*, *ALB*, *CYP1A2*, and *CYP3A4*). Expression levels were normalized to *GAPDH* and compared to the expression levels of hUC-MSCs on differentiation day 0. Two-way analysis of variance (ANOVA) was used, and *P*-values of hLD-SCs were compared to those of hUC-MSCs on differentiation days 0, 7, 14, and 21. *P*-values < 0.05 were considered significant. n = 4. *, *p* < 0.05; **, *p* < 0.01; ***, *p* < 0.001.

As our two-step in vitro differentiation protocol showed hepatocyte-like morphologic changes of stem cells, we tracked the changes using molecular dynamics during differentiation. It is known that stem cells experience a stage-wise differentiation into hepatocyte-like cells, namely from pluripotent cells into definitive endoderm, hepatic progenitor, and, finally, matured hepatocyte-like cells [49,50]. The current study tracked the changes in the representative stage indicator genes during hepatic lineage development (Figure 3A).

As we could observe the obvious changes in cell morphology, we next analyzed the antibody-based expression of human albumin as a hepatocyte maturation marker. The protein expression of albumin was not significant on day 7. However, cells started to express albumin on day 14, and this expression increased up to day 21 (Figure 3B).

For gene expression analyses, the expression values of stage-wise hepatic differentiation-related genes were compared with the expression levels of primary human hepatocytes (PHHs). First, three endoderm stage markers were measured (Figure 3C). Interestingly, hLD-SCs expressed high levels of *GATA4*, an important gene for endoderm development, compared to hUC-MSCs through the entire differentiation sequence. The basal expression levels of *GATA4* in hLD-SCs were higher than that of PHH, though the expression levels of *GATA4* in hLD-SCs decreased during hepatic differentiation. Forkhead box protein A1 (*FOXA1*) expression was relatively high during the stem cell stage in hLD-SCs, but the difference faded on day 7. *FOXA2* expression showed no difference between hUC-MSCs and hLD-SCs on day 0, but it increased significantly in hUC-MSCs on day 14. However, the expression levels of *FOXA2* in hLD-SCs were relatively high on day 14 compared to day 0, but the difference was not substantial (Figure 3C). Notably, all differentiated hUC-MSCs and hLD-SCs could not reach the expression levels of *FOXA1* and *FOXA2* in PHHs during the hepatic differentiation sequence. Considering that it has been reported that PHHs naturally express FOXA1/2 during embryonic development and in adulthood [51], this might be caused by the difference between naturally produced PHHs and in vitro-differentiated hepatocyte-like cells.

Currently, hepatic progenitor cells are the most widely accepted promising regenerative sources for curing liver diseases [52]. Therefore, we accordingly measured the expression of liver progenitor cell-related genes [50]. Carboxypeptidase M (*CPM*) is known to be highly expressed in embryonic liver progenitor cells [53]. *CD133* is reported to be mainly expressed in cancer stem cells [54], but is also widely accepted as a hepatic progenitor marker [55]. The hepatic progenitor cell markers, *CPM* and *CD133,* were significantly increased on day 14 in both hLD-SCs but not in hUC-MSCs (Figure 3D). However, hUC-MSCs and hLD-SCs did not express epithelial cell adhesion molecule (*EpCAM*) during hepatic differentiation (Supplementary Figure S2A). *EpCAM* has been reported as a marker for liver progenitor cells while it is known to have diverse roles in cell proliferation and differentiation [56], but mature hepatocytes do not express *EpCAM* [57]. Further, a recent study has reported that *EpCAM* expression can inhibit hepatic maturation in vitro [58]. In the following measurement of cytokeratin 19 (*CK19*), an indicative marker for hepatic progenitor cells and biliary differentiation, only undifferentiated hUM-MSCs expressed relatively high *CK19*, which diminished during the differentiation (Supplementary Figure S2B). This observation is consistent with previous reports, which state that undifferentiated hUC-MSCs can express *CK19* during expansion [18] and that hLD-SCs do not express *CK19* [32]. The other known hepatic progenitor cell marker, SRY-related HMG box transcription factor 9 (*SOX9*), was increased on day 14 of differentiation in all hUC-MSCs and two hLD-SCs while hLD-SC 1 cells reached their highest levels. The expression of *SOX9* was rapidly decreased on day 21 in hLD-SC 1 and hLD-SC 2 cells, but hUC-MSC cells on day 21 remained at elevated levels (Supplementary Figure S2C). *SOX9* is a widely accepted marker for cholangiocyte differentiation. However, *SOX9*-expressing cells are considered to take on an important role in activating hepatic progenitor cells to mature into hepatocytes [59]. With these results interpreted altogether, *EpCAM* and *CK19* might have little effects on the current in vitro hepatic differentiation outcomes while *CPM*, *CD133*, and *SOX9* can be indicative markers for hepatic differentiation of hLD-SCs.

The hepatocyte maturation markers were gradually increased during differentiation in all hUC-MSCs and hLD-SCs (Figure 3E). For *HNF1A* and cytochrome P450 1A2 (*CYP1A2*), though hLD-SCs showed a consistent rise during differentiation, hUC-MSCs showed the highest expression. For *HNF4A*, hLD-SCs showed increased expression on day 14, a trend absent in hUC-MSCs. For alpha-fetoprotein (*AFP*), *ALB*, and cytochrome P450 3A4 (*CYP3A4*), hLD-SC 1 cells showed similar expression patterns to that of hUC-MSCs during differentiation. However, *CYP3A4* of hLD-SC 2 cells were not elevated during hepatic differentiation.

Overall, hLD-SCs hold hepatogenic potential with high basal expressions of the endoderm markers, *GATA4* and *FOXA1*. Two-step hepatic differentiation seems to fall in the middle of maturation on day 14 with high levels of hepatic progenitor-related genes, *CPM* and *CD133*. hLD-SCs showed a consistent rise in hepatocyte maturation-related markers during hepatic differentiation. Yet, hLD-SCs offered no advantages in hepatic maturation compared to hUC-MSCs, at least in vitro.

### 3.4. Mitochondrial Oxygen Consumption Rate (OCR) and Metabolic Profile Comparison

**Figure 4:**
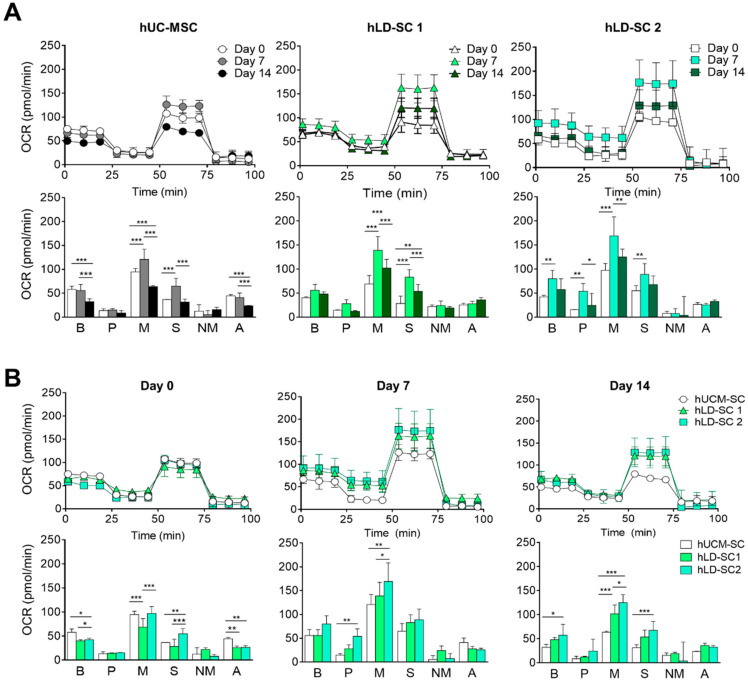
Mitochondrial oxygen consumption rates (OCRs) and biogenetic profiles of stem cells during hepatic differentiation. (**A**) OCRs during the seahorse assay and mitochondrial biogenic profiles of hUC-MSCs and two hLD-SCs on days 0, 7, and 14 of differentiation towards hepatic fate are presented. (**B**) OCRs during the seahorse assay and mitochondrial biogenic profiles on days 0, 7, and 14 of differentiation towards the hepatic fate of hUC-MSCs and two hLD-SCs are presented. B, basal respiration; P, proton leak; M, maximal respiration; S, spare respiratory capacity; NM, non-mitochondrial oxygen consumption; A, ATP production. *P*-values < 0.05 were considered significant. n = 4. *, *p* < 0.05; **, *p* < 0.01; ***, *p* < 0.001.

As the differentiation process requires high energy expenditure, recent studies have reported that mitochondrial function is important in differentiation processes, such as osteogenic differentiation [60] and adipogenesis [61]. OCR is a useful method to measure ATP production through oxidative phosphorylation (Oxphos) in mitochondria. Thus, we compared mitochondrial OCR during the two-step hepatic differentiation process. For hUC-MSCs and two hLD-SCs, OCR was increased on day 7 of differentiation. With respect to the metabolic profile, maximal respiration and spare respiratory capacity were significantly increased on day 7 of differentiation (Figure 4A). On day 0 of differentiation, hUC-MSCs and two hLD-SCs had similar OCR rates. Basal respiration and ATP production were slightly lower in the two hLD-SCs. However, on differentiation days 7 and 14, OCR, especially in terms of maximal respiration, was relatively high in the two hLD-SCs (Figure 4B). Taken together, these results suggest that the mitochondrial functions were similar when hUC-MSCs and hLD-SCs were undifferentiated, but when the cells undergo hepatic differentiation, hLD-SCs tend to exhibit a more favorable energy status. As we supplemented two small molecules, Fasudil and 5-azacytidine, to increase differentiation stability for the first 7 days, this might account for the increased OCR on day 7 of differentiation. Nevertheless, increased OCR in hLD-SCs does not seem to be related to hepatic differentiation outcome, especially considering that most hepatocyte maturation markers (*HFN1A*, *AFP*, and CYP family genes) were relatively low in differentiation-induced hLD-SCs.

### 3.5. Trafficking of hLD-SCs during Liver Ischemia-Reperfusion Injury (LIRI)

**Figure 5:**
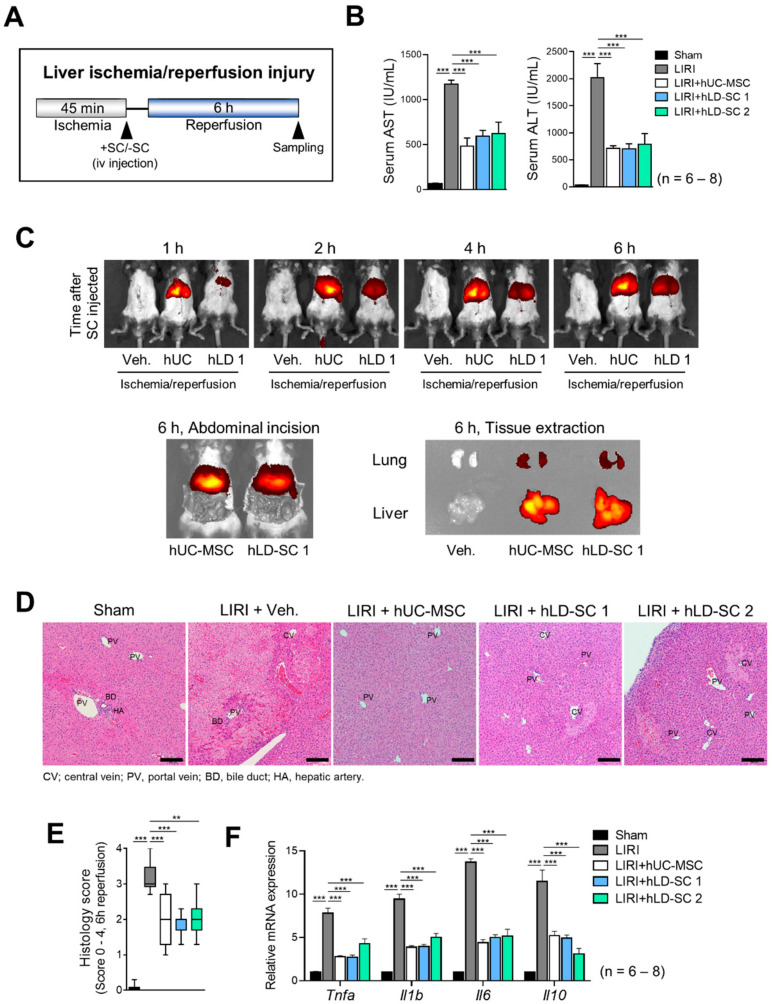
Trafficking of hUC-MSCs and hLD-SCs in liver ischemia-reperfusion injury (LIRI) mice. (**A**) Schematic diagram of LIRI in C57BL/6 mice. (**B**) Serum levels of aspartate transaminase (AST) and alanine transaminase (ALT). *P*-values < 0.05 were considered significant. n = 6 for sham; n = 8 for other groups. (**C**) hUC-MSCs and hLD-SCs were labeled with DiR fluorescent dye. Fluorescence signals were measured 1, 2, 4, and 6 h after administering hUC-MSCs or hLD-SCs. Fluorescence was measured after incising the abdomen or extracting the relevant tissues from each mouse. (**D**) Hematoxylin and eosin (H&E) staining for liver histology. Scale bar, 200 μm. (**E**) Suzuki score of each mouse. (**F**) Gene expression levels of pro-inflammatory cytokines (*Tnfa*, *Il1b*, and *Il6*) and anti-inflammatory cytokine, *Il10*. Expression levels were normalized to *GAPDH* and compared to the expression levels of the sham group. *P*-values < 0.05 were considered significant. n = 6 for sham; n = 8 for other groups. *, *p* < 0.05; **, *p* < 0.01; ***, *p* < 0.001.

MSCs are known to cause liver regeneration through their trophic nature and can protect livers from damage [17]. Besides these factors, exogenous MSCs are reported to translocate to organs with injuries [62]. As our in vitro studies revealed that hLD-SCs have MSC-like characteristics, we wondered what further characteristics can be observed in vivo. To measure cellular trafficking events, mice were exposed to liver ischemia for 45 min, then injected with each type of hUC-MSC or hLD-SCs intravenously via the tail vein, and then reperfused for 6 h (Figure 5A). The serum levels of AST and ALT were lower in mice treated with either hUC-MSCs or the two hLD-SCs compared to the untreated LIRI groups (Figure 5B). As the cells were labeled with the DiR fluorescent dye, we could measure the trafficking of cells using the IVIS Spectrum Imaging System. Following injection of the cells into mice via the tail vein, hUC-MSCs settled earlier than hLD-SCs. The density of fluorescent signals was higher in hUC-MSCs. This might be because of the size difference between hUC-MSCs and hLD-SCs. Representative images with abdominal incision and tissue extraction showed that stem cells injected via the tail vein were trafficked to the liver after 6 h (Figure 5C). The histology also showed consistent results such that hUC-MSCs and two hLD-SCs had a similar effect on reducing LIRI (Figure 5D,E). The mRNA levels of the inflammatory cytokines, *Tnfa*, *Il1b*, *Il6*, and *Il10* were all significantly increased in the untransplanted LIRI group, while stem cell treatment significantly attenuated this increase (Figure 5F).

### 3.6. Spontaneous Differentiation and Regeneration Potential of Differentiation-Induced hLD-SCs

**Figure 6:**
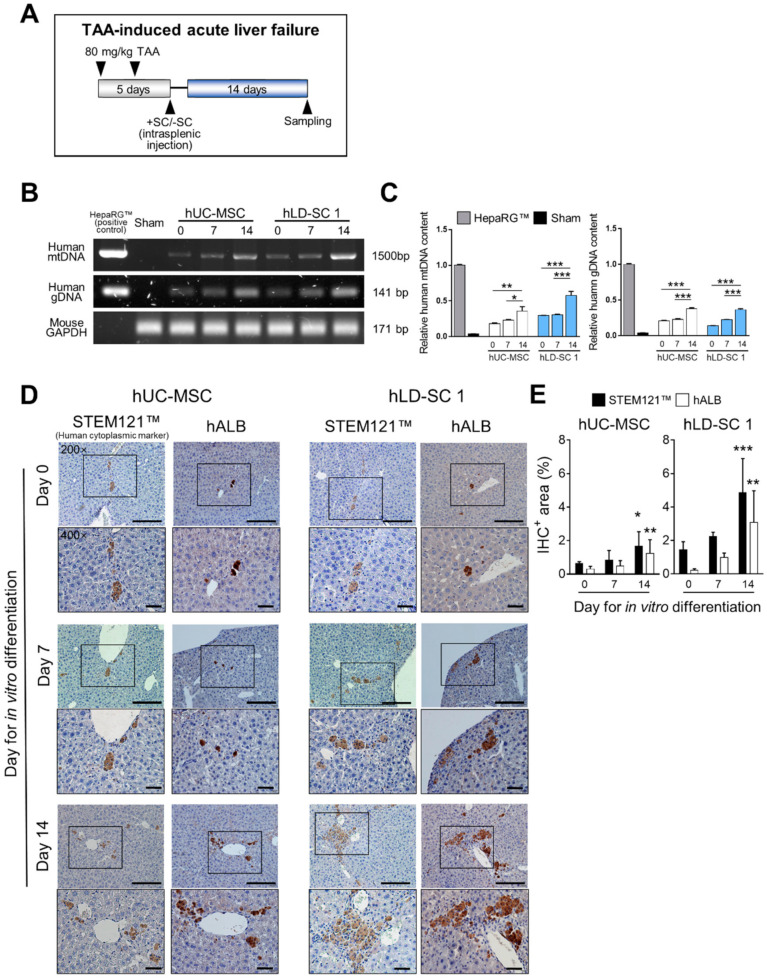
Spontaneous hepatocyte differentiation and maturation in thioacetamide (TAA)-induced acute liver failure mice. (**A**) Schematic diagram of TAA-induced acute liver injury in Rag2^-/-^Il2rg^-/-^ (NRG) mice. hUC-MSCs and hLD-SCs on days 0, 7, and 14 of differentiation towards hepatic fate were treated with intrasplenic administration, and mice were restored for 14 days. (**B**) Representative polymerase chain reaction (PCR) results of human mitochondrial DNA (human mtDNA; amplicon size, 1500 bp), human-specific genomic DNA (human gDNA; amplicon size, 141 bp), and mouse genomic *GAPDH* (Mouse *GAPDH*; amplicon size, 171 bp). Total DNA was isolated from paraffin blocks used for immunohistochemistry (IHC). (**C**) Quantification of the relative human mtDNA and gDNA contents. The contents were compared relative to the amount of mtDNA and gDNA of HepaRG™. Total DNA was extracted from 1 × 10^5^ HepaRG™ cells. (**D**) Representative images of human STEM121™ (human cytoplasmic marker) and human albumin (hALB) IHC in mice restored 14 days after stem cell transplantation. Scale bar, 100 μm for 200× magnification; Scale bar, 50 μm for 400× magnification. (**E**) STEM121™ and hALB positive area (%) of hUC-MSCs and hLD-SCs are expressed as mean ± standard deviation and each value was compared to day 0. *p*-values < 0.05 were considered significant. n = 3 for sham; n = 4 for other groups. *, *p* < 0.05; **, *p* < 0.01; ***, *p* < 0.001.

Our previous in vitro investigations suggested that hLD-SCs have distinct characteristics from hUC-MSCs with high basal endoderm marker expression (*GATA4* and *FOXA1*) and high hepatic progenitor cell-related gene expression on day 14 of differentiation (*CPM* and *CD133*), though no advantages from hepatocyte maturation-related markers were recognized. Based on these observations, we wondered whether hLD-SCs at different stages of in vitro differentiation show different outcomes after moving to an in vivo environment. To observe the in vivo response of hLD-SCs, immune-deficient NRG mice were treated with hepatotoxic TAA twice for five days, then hUC-MSCs or hLD-SCs were transplanted via the intrasplenic route. The mice were allowed to recover for 14 days (Figure 6A).

Mice on the fifth day showed a wide range of necrotic cell death and displayed typical symptoms of hepatic failure but mostly recovered after 14 days (Supplementary Figure S3A). The increased serum AST and ALT levels were also restored on day 14 (Supplementary Figure S3B). The same results were observed in hUC-MSCs or hLD-SCs from transplanted mice (Supplementary Figure S3C) with no meaningful changes in serum AST and ALT (Supplementary Figure S3D).

We could determine that human cells had trafficked to the liver of immune-deficient mice by measuring human-specific mitochondrial DNA (mtDNA) and genomic DNA (gDNA) amplicons (Figure 6B). The relative contents of mtDNA and gDNA were significantly high in the liver of mice transplanted with hepatic differentiation-induced cells compared to undifferentiated stem cells (Figure 6C). As our previous in vivo model showed hUC-MSCs and hLD-SCs trafficking into the liver when the damage occurred, the presence of human gDNA and mtDNA in the injured liver is quite reasonable.

To measure whether spontaneous differentiation and maturation into functional hepatocytes occurred in the liver, the human cell-specific cytoplasmic marker, STEM121™, was used to detect human cells, and human albumin (hALB) was detected as a human hepatocyte marker using IHC. The human cells and hALB-expressing cells were observed in all mice with cell transplantation (Figure 6D and Supplementary Figure S4A). We found that not all human cells remaining in the mice livers expressed hALB (Figure 6E and Supplementary Figure S4B). The 14-day differentiated cells showed advanced maturation with grape-like morphology of hALB-positive areas in hUC-MSCs and hLD-SC 1-transplanted mice. Particularly, 14 day-differentiated hLD-SC 1 cells, human cytoplasmic marker-positive cells, and hALB-positive cells were located mostly near intrahepatic bile ductules (also known as the canals of Hering). In hLD-SC 2 cell-transplanted mice, the cytoplasmic marker-positive cells were observed around the ductular areas, but lesser amounts of hALB-positive cells were observed compared to hLD-SC 1 cells. As in vitro hepatic differentiation of hLD-SC 2 cells exhibited relatively small changes, especially in hepatocyte maturation-related markers, those in vivo results are quite consistent.

Considering results together, hLD-SCs seem to have a liver-friendly nature, which makes them better able to settle within the in vivo environment. Though in vitro differentiation of hLD-SCs was incomplete with respect to the generation of hepatocyte maturation-related markers, when transplanted to the in vitro environment, they spontaneously differentiated to mature hepatocytes. Remarkably, when in vivo cell transplantation was combined with in vitro hepatic differentiation, hLD-SCs produced mature hepatocytes even faster than hUC-MSCs.

## 4. Discussion

hLD-SCs were evaluated as a possible source for liver regenerative therapy. However, the question remains whether adult stem cells originating from the liver can differentiate toward a hepatic lineage better than others. In the current study, we directly compared the differences of hLD-SCs and other types of adult stem cells during hepatic differentiation by comparing genes related to stage-wise hepatic differentiation and the OCR, which represents mitochondrial respiration. Lastly, we transplanted hepatic differentiation-induced hLD-SCs into two different mice models with liver injury.

We isolated hLD-SCs from three different liver donors using similar methods to those previously reported by Herrera [29] and Najimi [30]. Like hBM-MSCs or hUM-MSCs, hLD-SCs isolated in our study were positive for MSC markers (CD90 and CD105), while negative for the hematopoietic stem cell marker, CD34, and had definitive expression of the pluripotency markers, *OCT4*, *NANOG*, and *SOX2*. These results are consistent with prior studies reviewed by Kholodenko [35]. In terms of immunomodulation, hLD-SCs also manifested immunosuppressive properties by suppressing T cell proliferation during allogenic lymphocyte reactions. As cytokines expressed by MSCs are known to interact with resident cells after cell transplantation, we also compared the expression of representative cytokines such as *TNFα*, *IL1β*, *IL6*, *IL10*, and *TGFβ1*. However, the expression status varied in each cell type, and, therefore, it was difficult to define one distinctive characteristic. The differences seemed to be caused by donor differences because each donor had a different age, sex, and physiological status. In the current study, we could compare in vivo and in vitro responses of two different hLD-SCs from the livers of a 21-year-old male (hLD-SC 1) and 38-year-old female (hLD-SC 2). Though the pluripotency markers (*OCT4*, *NANOG*, and *SOX2*) were expressed highly in hLD-SC 2, in vitro and in vivo differentiation outcomes were better in hLD-SC 1 cells. As we do not understand which characteristics were attributed to dissimilar outcomes, further investigations of the association between donor characteristics and obtained hLD-SCs are needed.

Our one major question was based on the widely accepted hypothesis of whether MSCs emanating from a specific organ might be better suited for tissue repair and regeneration in the identical organ because they might already be accustomed to the innate microenvironments [35]. The current study sought to investigate whether hLD-SCs are more likely to differentiate into hepatocyte-like cells than MSCs originating from other sources, and if so, whether any key features exist. The in vitro differentiation of stem cells into hepatocyte-like cells followed a modified two-step differentiation protocol [19]. The Step-1 medium was supplemented with two small molecules; Fasudil, a RhoA/ROCK signaling inhibitor, and 5-azacytidine, a DNMT inhibitor. Fasudil was reported to suppress cell-cycle arrest and apoptosis in stem cells and was, therefore, widely used for maintaining various types of stem cells [63,64,65]. It has been previously observed that 5-azacytidine treatment inhibits global DNA methylation in MSCs, which helps to induce hepatocyte-specific gene expression [66]. Though we could not compare the difference between conventional differentiation and modified protocols with small molecules, we observed that our modified two-step hepatic differentiation protocol could differentiate both hUC-MSCs and hLD-SCs into hepatocyte-like cells. We could not compare the two protocols in parallel because cells without Fasudil and 5-azacytidine yielded inconsistent results with defective hepatic differentiation and were not appropriate for the current study. As such, we could conclude that two-step hepatic differentiation protocols combined with small molecules benefit hepatic differentiation.

We showed that hLD-SCs expressed higher levels of *GATA4* consistently through all stages of hepatocyte differentiation. As *GATA4* has been reported to be important for endoderm formation in stem cells [67], the high expression of *GATA4* in hLD-SCs suggests a high endodermal potency. *FOXA1* and *FOXA2* are known endoderm-specific markers [68]. The basal expression levels of *FOXA1* were high in hLD-SCs, while hLD-SCs and hUC-MSCs showed similar levels of *FOXA1* on day 7. Based on these findings, we concluded that hLD-SCs feature a more endoderm-like state compared to hUC-MSCs.

One interesting change during hepatocyte differentiation with hLD-SCs was the substantial increase in hepatic progenitor cell markers on day 14. Hepatic stellate cells with positive expression of *CD133* have been reported to be hepatic progenitor cells [69]. Further, *CPM* is reported to be a useful surface marker for human iPSC-derived hepatic progenitor cells [53]. These two markers were significantly (*P* < 0.001) increased on day 14 of differentiation in hLD-SCs but unchanged in hUC-MSCs. We also measured the expression of other known hepatic progenitor cell markers, *EpCAM*, *CK19*, and *SOX9*. However, *EpCAM* and *CK19* remained at very low levels during differentiation. Thus, based on *EpCAM* and *CK19* expression, our in vitro differentiation of hUC-MSCs and hLD-SCs might have happened independently. However, *SOX9* was elevated on day 14 of differentiation and decreased on day 21, showing consistency with previous reports [70]. hLD-SCs also showed an increase in hepatocyte maturation-related factors (*HNF1A*, *HNF4A*, and *AFP*) on day 14 of differentiation, but no further advantages were observed compared with hUC-MSCs. Based on these findings, we concluded that on day 14 of differentiation, hLD-SCs change into a hepatic progenitor-like state.

Interestingly, we found that the OCR of day 7-differentiated cells reached its highest levels, even in hLD-SCs. A previous study reported that OCR rises during hepatic maturation; however, the endoderm-like and hepatic progenitor-like states of cells did not lead to a significant increase in OCR [71]. In our study, we compared days 0, 7, and 14 of hepatic differentiation but not day 21-differentiated cells with a mature hepatocyte-like state. Nevertheless, the OCR of day 7-differentiated hUC-MSCs and hLD-SCs was increased significantly. This might be because of supplementation with small molecules. After removing small molecules, the increased OCRs decreased on day 14.

We also focused on mitochondrial respiration during differentiation. Functional changes in mitochondrial activity have been widely investigated during stem cell differentiation [72]. For example, during neural differentiation of human ESCs, a reduction in ATP demand and mitochondrial activity was observed [73]. In MSCs, the role of mitochondria in osteogenic, adipogenic, and chondrogenic differentiation has been investigated [74]. Energy metabolism is important for stem cell maintenance and differentiation because during differentiation into somatic cells, the Oxphos rate tends to increase [75]. In hepatic differentiation of ESCs, it was reported that the OCR tends to rise during hepatocyte maturation, and the OCR was highest in the late hepatocyte [76]. Our study compared days 0, 7, and 14 during hepatic differentiation, and OCR, especially maximal respiration and spare respiratory capacity, was notably elevated on day 7-differentiated cells in all hUC-MSCs and hLD-SCs. These results suggested that on day 7 of differentiation, induced stem cells might possess high differentiation potency, which enables the subsequent hepatic differentiation process.

In the current study, we also used two different in vivo models of liver injury. Both hUC-MSCs and hLD-SCs were trafficked to the injured liver within 2 h and suppressed liver injury in mice exposed to LIRI. As previous studies have shown that low dose *TNFα* can protect the liver from ischemia/reperfusion injury [77,78], the cytokines expressed in hUC-MSCs and hLD-SCs might help the liver suppress further stress. Recent reports show that hLD-SCs can also suppress hepatic stellate cell activation, which means protection against the development of cirrhotic liver [79]. It seems that hLD-SCs can be a therapeutic source for relieving acute liver injury.

TAA-induced acute liver failure model showed long-term engraftment of hLD-SCs in the liver. A five-day treatment of TAA was used to induce fulminant hepatic failure. As NRG mice tend to express more severe responses at standard concentrations of TAA (200–400 mg/kg) with early death, we adjusted the concentration of TAA to 80 mg/kg, and administration was reduced from every day to once every two days. This modification successfully helped keep the mice alive for up to 14 days after stem cell transplantation. A previous study demonstrated human cell trafficking in a liver hepatectomy mice model treated with hLD-SCs on days 7, 30, and 60 after transplantation [80]. Compared to that in a previous study, only 14-day-differentiated hLD-SCs showed similar hALB areas to naïve hLD-SCs maintained for 60 days in the liver. In addition, 14-day-differentiated hLD-SCs were trafficked near the intrahepatic bile ductules, which produced hepatic progenitor cells. This indicates the microenvironmental similarity of differentiated hLD-SCs and hepatic progenitor cells. Altogether, hepatic differentiation-induced hLD-SCs can settle within the liver and spontaneously mature into albumin-producing hepatocytes.

The strength of our study is the relatively easy handling and cost-effective maintenance of cells, simple and stable protocols for differentiation, and in vivo results that implicate spontaneous differentiation into mature hepatocytes in the liver after cell transplantation. However, as livers used in our study were obtained from healthy living donors for liver transplantation, we could not analyze hLD-SCs originating from diseased livers or livers from a deceased donor. As we failed to annotate specific relationships from donor-specific features of hLD-SCs, further studies are needed to compare different hLD-SCs isolated from livers of various statuses. In addition, we could observe many differences between isolated PHHs and in vitro differentiated hepatocyte-like cells. As various studies suggest the transcriptome differences between pluripotent cell-derived hepatocyte-like cells and naturally differentiated PHHs [71], it would be interesting to compare the whole transcriptome or proteome to discern the differences in the cultured and isolated cells.

**Figure 7:**
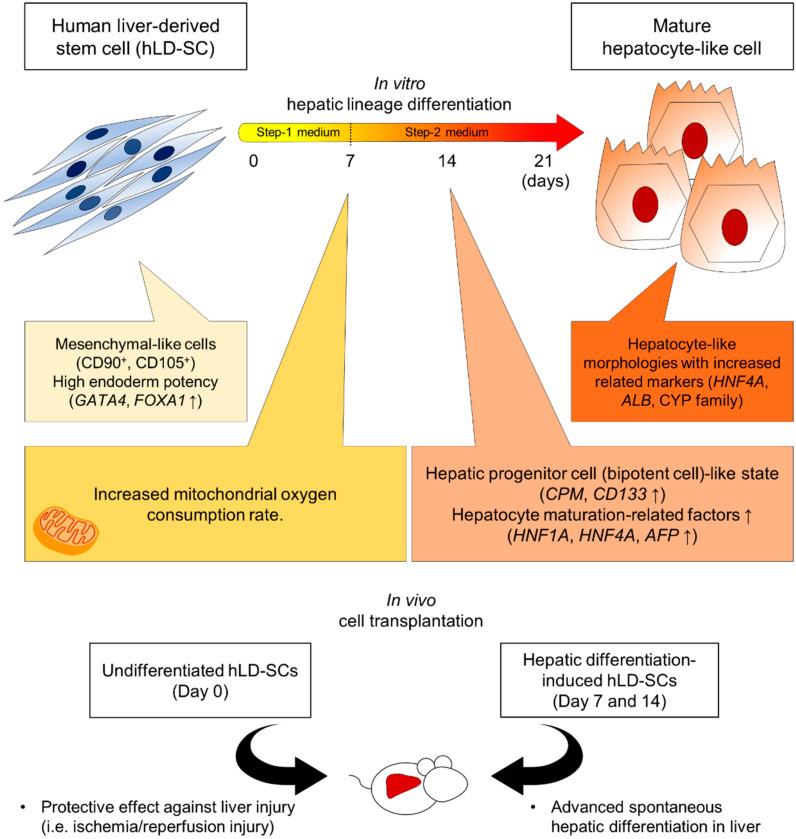
Schematic summary of in vitro characteristics during chemical-based hepatic differentiation. Human liver-derived stem cells (hLD-SCs) were pluripotent, expandable, and exhibited MSC-like structures with elongated spindle shape and ovoid nuclei. The hLD-SCs expressed high levels of endodermal genes (*GATA4* and *FOXA1*). On day 7 of the two-step chemical-based hepatic differentiation, hLD-SCs had a higher oxygen consumption rate (OCR), which related to the differentiation potency of MSCs. On day 14, hLD-SCs expressed high levels of the hepatic progenitor cell markers, *CPM* and *CD133*, with a consistent increase in hepatocyte maturation-related gene expression (*HNF1A*, *HNF4A*, and *AFP*). On day 21 of the two-step differentiation, hLD-SCs showed standard characteristics of mature hepatocyte-like cells with the conversion from a flat oval to a rough circle. In vivo cell transplantation of undifferentiated hLD-SCs exerted protective effects against liver injury and hepatic differentiation-induced hLD-SCs that were spontaneously differentiated into mature hepatocyte-like cells.

In summary, we hypothesized that stem cells originating from the liver would have better hepatic differentiation abilities than other tissue originated MSCs and compared the hepatocyte-differentiating effect between hUC-MSCs and hLD-SCs using our modified two-step hepatic differentiation protocol. In support of this hypothesis, hLD-SCs feature characteristics of high basal expression of *GATA4* and *FOXA1*, endoderm fate-related genes, and higher mitochondrial respiration on days 7 and 14 of differentiation, which suggests high energy potential as well as high expression of hepatic progenitor markers (*CPM* and *CD133*) on day 14 of differentiation in vitro. However, there were no further advantages in obtaining mature hepatocyte-like cells compared to differentiated hUC-MSCs. Nevertheless, in in vivo environments, hLD-SCs could traffic and settle within the injured liver and spontaneously differentiate into mature hepatocytes expressing hALB. Collectively, we posit that hLD-SCs hold better hepatogenic potential and may be a promising source for regenerative medicine (Figure 7).

## 5. Conclusions

hLD-SCs, mesenchymal-like stem cells obtained from human liver tissues, possess better hepatogenic differentiation ability with high basal levels of endoderm markers, increased hepatic progenitor cell-related gene expression on day 14 of differentiation, and advanced mitochondrial respiration during hepatic differentiation. In vivo cell transplantation showed that hepatic differentiation-induced hLD-SCs could traffic into the injured liver of mice and spontaneously differentiate into hepatocyte-like cells, suggesting the liver regenerative potential. A combination of in vitro differentiation and in vivo cell transplantation could suggest promising approaches for human liver-derived mesenchymal-like stem cell-based regenerative therapy.

## Figures and Tables

**Table 1:** Human liver donor information.

Liver Donor	1	2	3	Reference Range
Ethnicity	Asian	Asian	Asian	
Sex	Male	Female	Female	
Age (years)	21	38	27	
Body mass index	21.18	25.02	20.56	
Diagnosis	Living liverdonor	Living liverdonor	Living liverdonor	
Operation	Rightlobectomy	Rightlobectomy	Rightlobectomy	
Albumin (g/dL)	4.2	3.9	4.6	3.5–5.2
Total bilirubin (mg/dL)	0.2	0.4	0.7	0.2–1.2
AST (U/L)	20	20	21	< 40
ALT (U/L)	14	19	16	< 40
ABO group	O+	B+	B+	
HAV (COI)	Negative	Negative	Negative	Negative
HBV (U/mL)	Negative	Negative	Negative	Negative
HCV (COI)	Negative	Negative	Negative	Negative
Glucose (mg/dL)	94	89	94	70–99
Creatinine (mg/dL)	0.81	0.79	0.77	0.70–1.40
BUN (mg/dL)	12	8 *	10	10–26
Uric acid (mg/dL)	6.7	4.6	5.4	3–7
Total cholesterol (mg/dL)	146	140	235 *	< 199
Total protein (g/dL)	7.1	7.0	7.7	6–8
Alkaline phosphatase (U/L)	73	63	58	40–120
Gamma-GT (U/L)	13	19	17	5–36
Triglyceride (mg/dL)	51	157	56	< 199
HDL-cholesterol (mg/dL)	66	52	90 *	40 <

* These values are outside of the reference range.

**Table 2:** Primer information.

Gene Name	Primer Sequences (5′ to 3′)
Human	
*OCT4*	Forward: GAAGGATGTGGTCCGAGTGT Reverse: GTGAAGTGAGGGCTCCCATA
*NANOG*	Forward: CAAAGGCAAACAACCCACTT Reverse: TCTGCTGGAGGCTGAGGTAT
*SOX2*	Forward: AACCCCAAGATGCACAACTC Reverse: CGGGGCCGGTATTTATAATC
*TNFα*	Forward: CAGAGGGCCTGTACCTCATC Reverse: GGAAGACCCCTCCCAGATAG
*IL1* *β*	Forward: GGGCCTCAAGGAAAAGAATC Reverse: TTCTGCTTGAGAGGTGCTGA
*IL6*	Forward: TACCCCCAGGAGAAGATTCC Reverse: TTTTCTGCCAGTGCCTCTTT
*TGF* *β*	Forward: GCCATTTAATGGCAATGGTAGTCT Reverse: CACAGGGAGCTTGCAGAGAT
*IL10*	Forward: TGCCTTCAGCAGAGTGAAGA Reverse: GGTCTTGGTTCTCAGCTTGG
*GATA4*	Forward: GGCCTGTCATCTCACTACGG Reverse: ATGGCCAGACATCGCACT
*FOXA1*	Forward: GACTCCAGCCTCCTCAACTG Reverse: CTCCGAGGAGGACATGAGGT
*FOXA2*	Forward: CTTCAAGCACCTGCAGATTC Reverse: AGACCTGGATTTCACCGTGT
*CPM*	Forward: GGATGGAAGCGTTTTTGAAG Reverse: CCACAACAAGAACCCACAGG
*CD133*	Forward: AAGCATTGGCATCTTCTATGG Reverse: AAGCACAGAGGGTCATTGAGA
*HNF1A*	Forward: TGGGTCCTACGTTCACCAAC Reverse: TCTGCACAGGTGGCATGAGC
*HNF4A*	Forward: CAGGCTCAAGAAATGCTTCC Reverse: GGCTGCTGTCCTCATAGCTT
*AFP*	Forward: AGACTGCTGCAGCCAAAGTGA Reverse: GTGGGATCGATGCTGGAGTG
*ALB*	Forward: TGCTGATGAGTCAGCTGAAAA Reverse: TCAGCCATTTCACCATAGGTT
*CYP1A2*	Forward: CTTCGTAAACCAGTGGCAGG Reverse: AGGGCTTGTTAATGGCAGTG
*CYP3A4*	Forward: TTTTGTCCTACCATAAGGGCTTT Reverse: CACAGGCTGTTGACCATCAT
*GAPDH*	Forward: GCCTCAAGATCATCAGCAATGC Reverse: TGGTCATGAGTCCTTCCACGAT
Human mitochondrial DNA	Forward: CCCAAGACAACCAACCAAAA Reverse: ACTAGCTTATATGCTTGGGG
Human genomic DNA	Forward: ATGCTGATGTCTGGGTAGGGTG Reverse: TGAGTCAGGAGCCAGCGTATG
**Mouse**
*Tnfa*	Forward: AGCCCCCAGTCTGTATCCTT Reverse: CTCCCTTTGCAGAACTCAGG
*Il1b*	Forward: GCCCATCCTCTGTGACTCAT Reverse: AGGCCACAGGTATTTTGTCG
*Il6*	Forward: AGTTGCCTTCTTGGGACTGA Reverse: TCCACGATTTCCCAGAGAAC
*Il10*	Forward: CCAAGCCTTATCGGAAATGA Reverse: TTTTCACAGGGGAGAAATCG
GAPDH	Forward: AGGTCGGTGTGAACGGATTTG Reverse: TGTAGACCATGTAGTTGAGGTCA
GAPDH genomic DNA	Forward: ACCCAGAAGACTGTGGATGG Reverse: CACATTGGGGGTAGGAACAC

**Table 3:** Suzuki scoring scale.

Score	Congestion	Cytoplasmic Vacuolization	Parenchymal Necrosis
0	No	No	No
1	Minimal	Minimal	Single-cell necrosis
2	Mild	Mild	<30%
3	Moderate	Moderate	<60%
4	Severe	Severe	>60%

## Supplementary Materials

The following are available online at https://www.mdpi.com/2073-4409/9/6/1521/s1, Figure S1: Flow cytometry histogram profiles of CD34-positive cells, Figure S2: Relative normalized expression of *EpCAM*, *CK19*, and *SOX9*, Figure S3: Thioacetamide (TAA)-induced acute liver injury was mostly recovered within 14 days, Figure S4: Observation of spontaneous hepatocyte differentiation and maturation of hLD-SC 2 in thioacetamide (TAA)-induced acute liver injury mice, Table S1: Primer details for *EpCAM*, *CK19*, and *SOX9*.

## References

[1] Keeffe E.B. (2001). Liver transplantation: Current status and novel approaches to liver replacement. Gastroenterology.

[2] Moon D.B., Lee S.G. (2009). Liver transplantation. Gut Liver.

[3] Lantieri L., Grimbert P., Ortonne N., Suberbielle C., Bories D., Gil-Vernet S., Lemogne C., Bellivier F., Lefaucheur J.P., Schaffer N. (2016). Face transplant: Long-term follow-up and results of a prospective open study. Lancet.

[4] Burra P., Tomat S., Villa E., Gasbarrini A., Costa A.N., Conconi M.T., Forbes S.J., Farinati F., Cozzi E., Alison M.R. (2008). Experimental hepatology applied to stem cells. Dig. Liver Dis..

[5] Tanaka K., Soto-Gutierrez A., Navarro-Alvarez N., Rivas-Carrillo J.D., Jun H.S., Kobayashi N. (2006). Functional hepatocyte culture and its application to cell therapies. Cell Transplant..

[6] Denker H.W. (2006). Potentiality of embryonic stem cells: An ethical problem even with alternative stem cell sources. J. Med. Eth..

[7] Yu Y., Wang X., Nyberg S.L. (2014). Potential and Challenges of Induced Pluripotent Stem Cells in Liver Diseases Treatment. J. Clin. Med..

[8] Meirelles L.D.S., Chagastelles P.C., Nardi N.B. (2006). Mesenchymal stem cells reside in virtually all post-natal organs and tissues. J. Cell Sci..

[9] Tsai C.C., Hung S.C. (2012). Functional roles of pluripotency transcription factors in mesenchymal stem cells. Cell Cycle.

[10] Aggarwal S., Pittenger M.F. (2005). Human mesenchymal stem cells modulate allogeneic immune cell responses. Blood.

[11] Kuo T.K., Ho J.H., Lee O.K. (2009). Mesenchymal stem cell therapy for nonmusculoskeletal diseases: Emerging applications. Cell Transplant..

[12] Pittenger M.F., Mackay A.M., Beck S.C., Jaiswal R.K., Douglas R., Mosca J.D., Moorman M.A., Simonetti D.W., Craig S., Marshak D.R. (1999). Multilineage potential of adult human mesenchymal stem cells. Science.

[13] Fu X., Liu G., Halim A., Ju Y., Luo Q., Song A.G. (2019). Mesenchymal Stem Cell Migration and Tissue Repair. Cells.

[14] Burra P., Tomat S., Bizzaro D., Pellicanò D., Masier A., Conconi M.T., Russo F., Bo P., Parnigotto P.P. (2008). Stem cells in hepatology. Dig. Liver Dis..

[15] van Poll D., Parekkadan B., Cho C.H., Berthiaume F., Nahmias Y., Tilles A.W., Yarmush M.L. (2008). Mesenchymal stem cell-derived molecules directly modulate hepatocellular death and regeneration in vitro and in vivo. Hepatology.

[16] Wang Y.H., Wu D.B., Chen B., Chen E.Q., Tang H. (2018). Progress in mesenchymal stem cell-based therapy for acute liver failure. Stem Cell Res. Ther..

[17] Francois S., Mouiseddine M., Allenet-Lepage B., Voswinkel J., Douay L., Benderitter M., Chapel A. (2013). Human mesenchymal stem cells provide protection against radiation-induced liver injury by antioxidative process, vasculature protection, hepatocyte differentiation, and trophic effects. BioMed Res. Int..

[18] Campard D., Lysy P.A., Najimi M., Sokal E.M. (2008). Native umbilical cord matrix stem cells express hepatic markers and differentiate into hepatocyte-like cells. Gastroenterology.

[19] Lee K.D., Kuo T.K., Whang-Peng J., Chung Y.F., Lin C.T., Chou S.H., Chen J.R., Chen Y.P., Lee O.K. (2004). In vitro hepatic differentiation of human mesenchymal stem cells. Hepatology.

[20] Zhang Y., Li Y., Zhang L., Li J., Zhu C. (2018). Mesenchymal stem cells: Potential application for the treatment of hepatic cirrhosis. Stem Cell Res. Ther..

[21] Tan E.K., Shuh M., Francois-Vaughan H., Sanders J.A., Cohen A.J. (2017). Negligible Oval Cell Proliferation Following Ischemia-Reperfusion Injury With and Without Partial Hepatectomy. Ochsner J..

[22] Fausto N. (2004). Liver regeneration and repair: Hepatocytes, progenitor cells, and stem cells. Hepatology.

[23] Shupe T.D., Piscaglia A.C., Oh S.H., Gasbarrini A., Petersen B.E. (2009). Isolation and characterization of hepatic stem cells, or “oval cells,” from rat livers. Methods Mol. Biol..

[24] Li J., Xin J., Zhang L., Wu J., Jiang L., Zhou Q., Li J., Guo J., Cao H., Li L. (2014). Human hepatic progenitor cells express hematopoietic cell markers CD45 and CD109. Int. J. Med. Sci..

[25] Kim Y., Kang K., Lee S.B., Seo D., Yoon S., Kim S.J., Jang K., Jung Y.K., Lee K.G., Factor V.M. (2019). Small molecule-mediated reprogramming of human hepatocytes into bipotent progenitor cells. J. Hepatol..

[26] Xie B.Q., Sun D., Du Y.Y., Jia J., Sun S.C., Xu J., Liu Y.F., Xiang C.G., Chen S.T., Xie H.F. (2019). A two-step lineage reprogramming strategy to generate functionally competent human hepatocytes from fibroblasts. Cell Res..

[27] Zhang K., Zhang L., Liu W., Ma X., Cen J., Sun Z., Wang C., Feng S., Zhang Z., Yue L. (2018). In Vitro Expansion of Primary Human Hepatocytes with Efficient Liver Repopulation Capacity. Cell Stem Cell.

[28] Li X., Liu D.F., Ma Y.T., Du X.M., Jing J.Z., Wang L.P., Xie B.Q., Sun D., Sun S.Q., Jin X.Q. (2017). Direct Reprogramming of Fibroblasts via a Chemically Induced XEN-like State. Cell Stem Cell.

[29] Herrera M.B., Bruno S., Buttiglieri S., Tetta C., Gatti S., Deregibus M.C., Bussolati B., Camussi G. (2006). Isolation and characterization of a stem cell population from adult human liver. Stem Cells.

[30] Najimi M., Khuu D.N., Lysy P.A., Jazouli N., Abarca J., Sempoux C., Sokal E.M. (2007). Adult-derived human liver mesenchymal-like cells as a potential progenitor reservoir of hepatocytes?. Cell Transplant..

[31] Pan Q.W., Fouraschen S.M.G., Kaya F.S.F.A., Verstegen M.M., Pescatori M., Stubbs A.P., van IJcken W., van der Sloot A., Smits R., Kwekkeboom J. (2011). Mobilization of Hepatic Mesenchymal Stem Cells From Human Liver Grafts. Liver Transplant..

[32] Lee J.H., Park H.J., Kim Y.A., Lee D.H., Noh J.K., Kwon C.H.D., Jung S.M., Lee S.K. (2012). The Phenotypic Characteristic of Liver-Derived Stem Cells From Adult Human Deceased Donor Liver. Transplant. Proc..

[33] Luo X.B., Gupta K., Ananthanarayanan A., Wang Z.N., Xia L., Li A.M., Sakban R.B., Liu S.D., Yu H. (2018). Directed Differentiation of Adult Liver Derived Mesenchymal Like Stem Cells into Functional Hepatocytes. Sci. Rep..

[34] Wang Y., Yu X., Chen E., Li L. (2016). Liver-derived human mesenchymal stem cells: A novel therapeutic source for liver diseases. Stem Cell Res. Ther..

[35] Kholodenko I.V., Kurbatov L.K., Kholodenko R.V., Manukyan G.V., Yarygin K.N. (2019). Mesenchymal Stem Cells in the Adult Human Liver: Hype or Hope?. Cells.

[36] Li W., Li K., Wei W., Ding S. (2013). Chemical approaches to stem cell biology and therapeutics. Cell Stem Cell.

[37] Song H., Chang W., Song B.W., Hwang K.C. (2012). Specific differentiation of mesenchymal stem cells by small molecules. Am. J. Stem Cells.

[38] Korostylev A., Mahaddalkar P.U., Keminer O., Hadian K., Schorpp K., Gribbon P., Lickert H. (2017). A high-content small molecule screen identifies novel inducers of definitive endoderm. Mol. Metab..

[39] So S., Lee Y., Choi J., Kang S., Lee J.Y., Hwang J., Shin J., Dutton J.R., Seo E.J., Lee B.H. (2020). The Rho-associated kinase inhibitor fasudil can replace Y-27632 for use in human pluripotent stem cell research. PLoS ONE.

[40] He Y., Cui J., He T., Bi Y. (2015). 5-azacytidine promotes terminal differentiation of hepatic progenitor cells. Mol. Med. Rep..

[41] Kim J., Piao Y., Pak Y.K., Chung D., Han Y.M., Hong J.S., Jun E.J., Shim J.-Y., Choi J., Kim C.J. (2014). Umbilical cord mesenchymal stromal cells affected by gestational diabetes mellitus display premature aging and mitochondrial dysfunction. Stem Cells Dev..

[42] Kim H., Yang G., Park J., Choi J., Kang E., Lee B.K. (2019). Therapeutic effect of mesenchymal stem cells derived from the human umbilical cord in a rabbit temporomandibular joint model of osteoarthritis. Sci. Rep..

[43] Park B.-W., Kang E.-J., Byun J.-H., Son M.-G., Kim H.-J., Hah Y.-S., Kim T.-H., Mohana Kumar B., Ock S.-A., Rho G.-J. (2012). In vitro and in vivo osteogenesis of human mesenchymal stem cells derived from skin, bone marrow and dental follicle tissues. Differentiation.

[44] Kang E.J., Wang X.J., Tippner-Hedges R., Ma H., Folmes C.D.L., Gutierrez N.M., Lee Y.M., Van Dyken C., Ahmed R., Li Y. (2016). Age-Related Accumulation of Somatic Mitochondrial DNA Mutations in Adult-Derived Human iPSCs. Cell Stem Cell.

[45] Suzuki S., Toledo-Pereyra L.H., Rodriguez F.J., Cejalvo D. (1993). Neutrophil infiltration as an important factor in liver ischemia and reperfusion injury. Transplantation.

[46] Kyurkchiev D., Bochev I., Ivanova-Todorova E., Mourdjeva M., Oreshkova T., Belemezova K., Kyurkchiev S. (2014). Secretion of immunoregulatory cytokines by mesenchymal stem cells. World J. Stem Cells.

[47] Klein S.L., Flanagan K.L. (2016). Sex differences in immune responses. Nat. Rev. Immunol..

[48] Bloom D.D., Centanni J.M., Bhatia N., Emler C.A., Drier D., Leverson G.E., McKenna D.H., Gee A.P., Lindblad R., Hei D.J. (2015). A reproducible immunopotency assay to measure mesenchymal stromal cell-mediated T-cell suppression. Cytotherapy.

[49] Meseguer-Ripolles J., Khetani S.R., Blanco J.G., Iredale M., Hay D.C. (2018). Pluripotent Stem Cell-Derived Human Tissue: Platforms to Evaluate Drug Metabolism and Safety. AAPS J..

[50] Chaudhari P., Tian L., Deshmukh A., Jang Y.Y. (2016). Expression kinetics of hepatic progenitor markers in cellular models of human liver development recapitulating hepatocyte and biliary cell fate commitment. Exp. Biol. Med..

[51] Lau H.H., Ng N.H.J., Loo L.S.W., Jasmen J.B., Teo A.K.K. (2018). The molecular functions of hepatocyte nuclear factors - In and beyond the liver. J. Hepatol..

[52] Tsuchiya A., Lu W.Y. (2019). Liver stem cells: Plasticity of the liver epithelium. World J. Gastroenterol..

[53] Kido T., Koui Y., Suzuki K., Kobayashi A., Miura Y., Chern E.Y., Tanaka M., Miyajima A. (2015). CPM Is a Useful Cell Surface Marker to Isolate Expandable Bi-Potential Liver Progenitor Cells Derived from Human iPS Cells. Stem Cell Rep..

[54] Mizrak D., Brittan M., Alison M.R. (2008). CD133: Molecule of the moment. J. Pathol..

[55] Zhao D.X., Chen S., Cai J., Guo Y.S., Song Z.H., Che J., Liu C., Wu C., Ding M.X., Deng H.K. (2009). Derivation and Characterization of Hepatic Progenitor Cells from Human Embryonic Stem Cells. PLoS ONE.

[56] Okabe M., Tsukahara Y., Tanaka M., Suzuki K., Saito S., Kamiya Y., Tsujimura T., Nakamura K., Miyajima A. (2009). Potential hepatic stem cells reside in EpCAM+ cells of normal and injured mouse liver. Development.

[57] Gires O. (2012). EpCAM in hepatocytes and their progenitors. J. Hepatol..

[58] Tang D., Chen Y., Fu G.B., Yuan T.J., Huang W.J., Wang Z.Y., Li W.J., Jiao Y.F., Yu W.F., Yan H.X. (2020). EpCAM inhibits differentiation of human liver progenitor cells into hepatocytes in vitro by activating Notch1 signaling. Biochem. Biophys. Res. Commun..

[59] Furuyama K., Kawaguchi Y., Akiyama H., Horiguchi M., Kodama S., Kuhara T., Hosokawa S., Elbahrawy A., Soeda T., Koizumi M. (2011). Continuous cell supply from a Sox9-expressing progenitor zone in adult liver, exocrine pancreas and intestine. Nat. Genet..

[60] Shen Y.L., Wu L., Qin D.D., Xia Y.K., Zhou Z., Zhang X.M., Wu X. (2018). Carbon black suppresses the osteogenesis of mesenchymal stem cells: The role of mitochondria. Part. Fibre Toxicol..

[61] Forni M.F., Peloggia J., Trudeau K., Shirihai O., Kowaltowski A.J. (2016). Murine Mesenchymal Stem Cell Commitment to Differentiation Is Regulated by Mitochondrial Dynamics. Stem Cells.

[62] Ullah M., Liu D.D., Thakor A.S. (2019). Mesenchymal Stromal Cell Homing: Mechanisms and Strategies for Improvement. iScience.

[63] Olson M.F. (2008). Applications for ROCK kinase inhibition. Curr. Opin. Cell Biol..

[64] Ding J., Li Q.Y., Yu J.Z., Wang X., Sun C.H., Lu C.Z., Xiao B.G. (2010). Fasudil, a Rho kinase inhibitor, drives mobilization of adult neural stem cells after hypoxia/reoxygenation injury in mice. Mol. Cell. Neurosci..

[65] Tsuno A., Nasu K., Kawano Y., Yuge A., Li H.L., Abe W., Narahara H. (2011). Fasudil Inhibits the Proliferation and Contractility and Induces Cell Cycle Arrest and Apoptosis of Human Endometriotic Stromal Cells: A Promising Agent for the Treatment of Endometriosis. J. Clin. Endocrinol. Metab..

[66] Lee C.-W., Huang W.-C., Huang H.-D., Huang Y.-H., Ho J.H., Yang M.-H., Yang V.W., Lee O.K. (2017). DNA methyltransferases modulate hepatogenic lineage plasticity of mesenchymal stromal cells. Stem Cell Rep..

[67] Fisher J.B., Pulakanti K., Rao S., Duncan S.A. (2017). GATA6 is essential for endoderm formation from human pluripotent stem cells. Biol. Open.

[68] Wang P., McKnight K.D., Wong D.J., Rodriguez R.T., Sugiyama T., Gu X.Y., Ghodasara A., Qu K., Chang H.Y., Kim S.K. (2012). A Molecular Signature for Purified Definitive Endoderm Guides Differentiation and Isolation of Endoderm from Mouse and Human Embryonic Stem Cells. Stem Cells Dev..

[69] Kordes C., Sawitza I., Muller-Marbach A., Ale-Agha N., Keitel V., Klonowski-Stumpe H., Haussinger D. (2007). CD133(+) hepatic stellate cells are progenitor cells. Biochem. Biophys. Res. Commun..

[70] Van Eyken P., Sciot R., Callea F., Van der Steen K., Moerman P., Desmet V.J. (1988). The development of the intrahepatic bile ducts in man: A keratin-immunohistochemical study. Hepatology.

[71] Wruck W., Adjaye J. (2018). Human pluripotent stem cell derived HLC transcriptome data enables molecular dissection of hepatogenesis. Sci. Data.

[72] Wanet A., Arnould T., Najimi M., Renard P. (2015). Connecting Mitochondria, Metabolism, and Stem Cell Fate. Stem Cells Dev..

[73] Birket M.J., Orr A.L., Gerencser A.A., Madden D.T., Vitelli C., Swistowski A., Brand M.D., Zeng X.M. (2011). A reduction in ATP demand and mitochondrial activity with neural differentiation of human embryonic stem cells. J. Cell Sci..

[74] Li Q.Q., Gao Z.W., Chen Y., Guan M.X. (2017). The role of mitochondria in osteogenic, adipogenic and chondrogenic differentiation of mesenchymal stem cells. Protein Cell.

[75] Hu C.X., Fan L.X., Cen P.P., Chen E.M., Jiang Z.Y., Li L.J. (2016). Energy Metabolism Plays a Critical Role in Stem Cell Maintenance and Differentiation. Int. J. Mol. Sci..

[76] Hopkinson B.M., Desler C., Kalisz M., Vestentoft P.S., Rasmussen L.J., Bisgaard H.C. (2017). Bioenergetic Changes during Differentiation of Human Embryonic Stem Cells along the Hepatic Lineage. Oxid. Med. Cell. Longev..

[77] Abu-Amara M., Yang S.Y., Tapuria N., Fuller B., Davidson B., Seifalian A. (2010). Liver ischemia/reperfusion injury: Processes in inflammatory networks—A review. Liver Transplant..

[78] Teoh N., Leclercq I., Pena A.D., Farrell G. (2003). Low-dose TNF-α protects against hepatic ischemia-reperfusion injury in mice: Implications for preconditioning. Hepatology.

[79] Najimi M., Berardis S., El-Kehdy H., Rosseels V., Evraerts J., Lombard C., El Taghdouini A., Henriet P., van Grunsven L., Sokal E.M. (2017). Human liver mesenchymal stem/progenitor cells inhibit hepatic stellate cell activation: In vitro and in vivo evaluation. Stem Cell Res. Ther..

[80] Khuu D.N., Nyabi O., Maerckx C., Sokal E., Najimi M. (2013). Adult Human Liver Mesenchymal Stem/Progenitor Cells Participate in Mouse Liver Regeneration After Hepatectomy. Cell Transplant..

