# Supplementary material for: Hepatogenic Potential and Liver Regeneration Effect of Human Liver-derived Mesenchymal-Like Stem Cells
Source: Cells. 2020 Jun 22;9(6):1521. doi: 10.3390/cells9061521 (PMC7348751; doi:10.3390/cells9061521)
Supplement: Supplementary file 1 [file cells-09-01521-s001.pdf]

cells

MDPI

Supplementary Data

1. Supplementary Figures

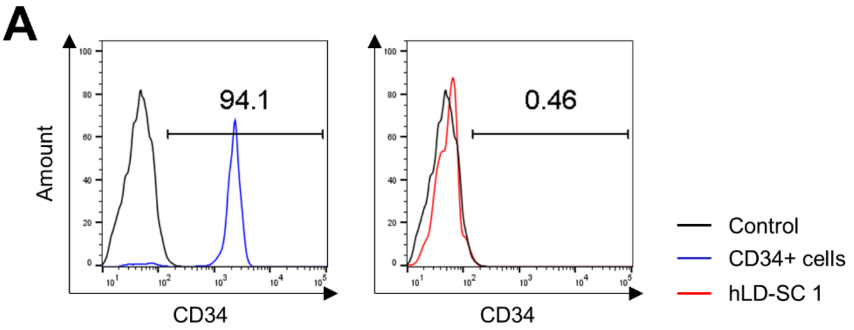

Figure S1. Flow cytometry histogram profiles of CD34-positive cells. (A) Flow cytometry measurement of CD34-positive cells and hLD-SC 1.

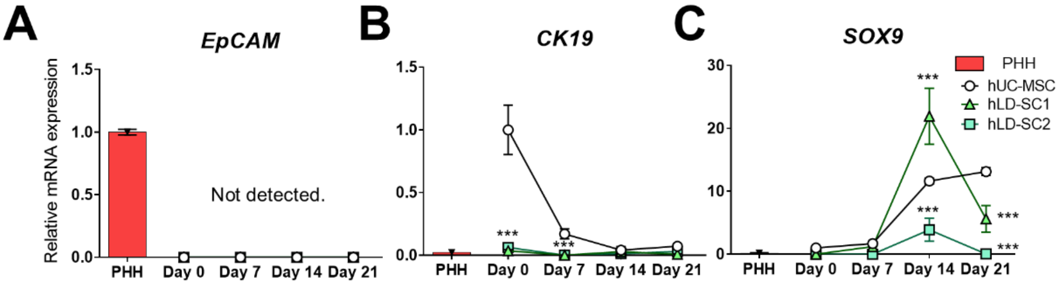

Figure S2. Relative normalized expression of *EpCAM*, *CK19*, and *SOX9*. (A – C) Expression levels of (A) *EpCAM*, (B) *CK19*, and (C) *SOX9* were normalized to *GAPDH* and compared to the expression levels of hUC-MSCs on differentiation day 0. Two-way analysis of variance (ANOVA) was used for statistical analysis, and *P*-values of hLD-SCs were compared to hUC-MSCs on differentiation days 0, 7, 14, and 21. *p*-values with < 0.05 were considered significant. n = 4. *, $p < 0.05$; **, $p < 0.01$; ***, $p < 0.001$.

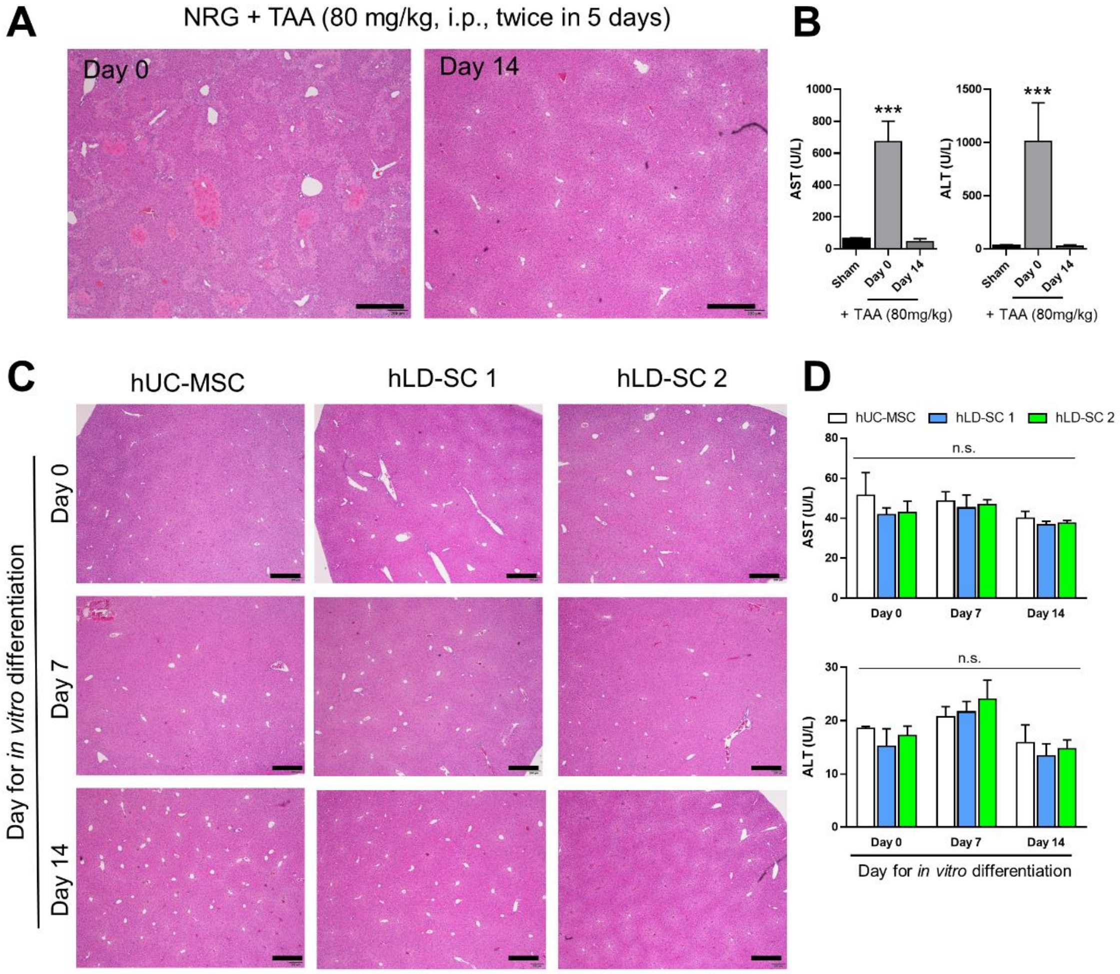

Figure S3. Thioacetamide (TAA)-induced acute liver injury was mostly recovered within 14 days. (A) Hematoxylin and eosin (H&E) staining for liver histology. 40× magnification; Scale bar, 500 μm. (B) Serum levels of aspartate transaminase (AST) and alanine transaminase (ALT) on day 0 and day 14 after TAA (80 mg/kg) administration. *P*-values < 0.05 were considered significant. n= 3. ***, $p < 0.001$. (C) H&E staining results for hUC-MSC- and hLD-SC-transplanted mice on day 14. 40× magnification; Scale bar, 500 μm. (D) Serum levels of AST and ALT on day 14 after cell transplantation. *p*-values < 0.05 were considered significant. n = 3 for sham; n = 4 for other groups. n.s., not significant.

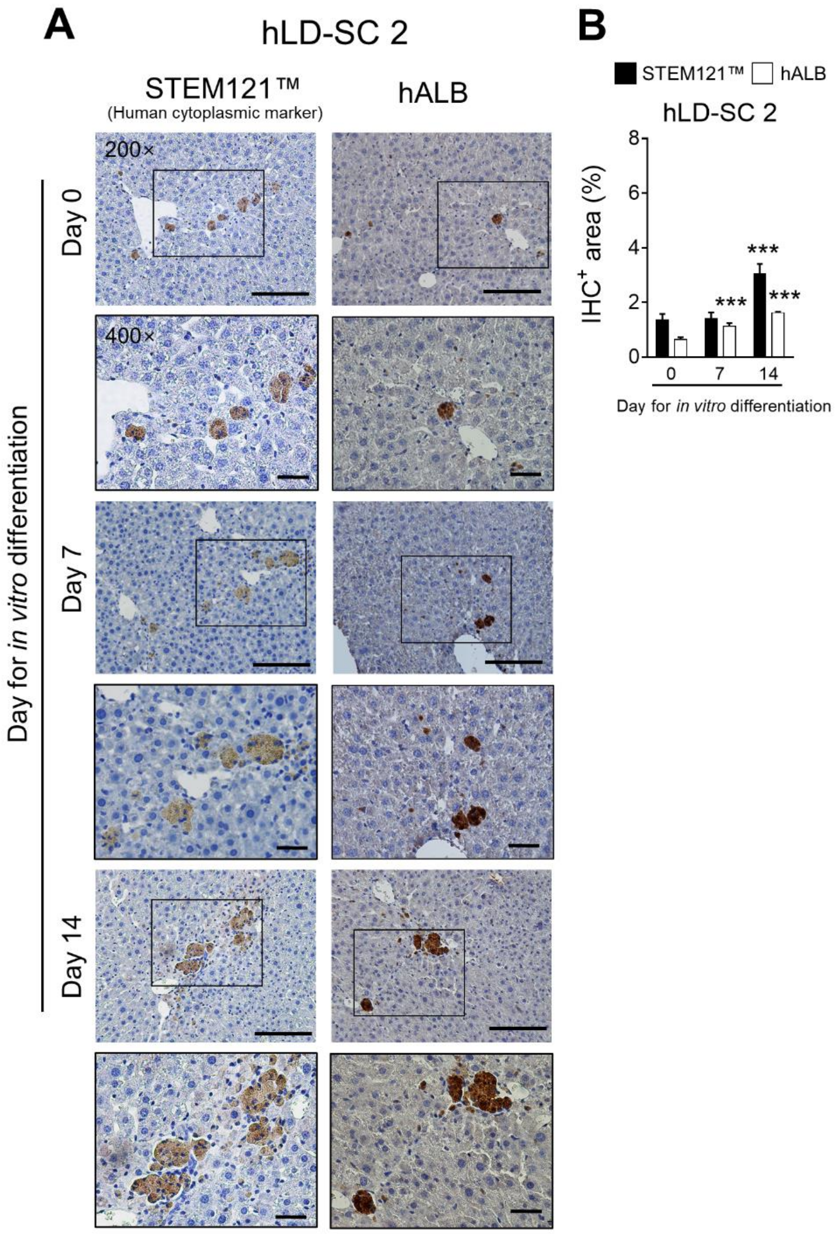

Figure S4. Observation of spontaneous hepatocyte differentiation and maturation of hLD-SC 2 in thioacetamide (TAA)-induced acute liver injury mice. (A) Representative images of human STEM121™ (human cytoplasmic marker) and human albumin (hALB) immunohistochemistry in mice restored 14 days after stem cell transplantation. Scale bar, 100 μm for 200× magnification; 50 μm for 400× magnification. (B) STEM121™ and hALB-positive area (%) of hUC-MSCs and hLD-SCs are expressed as average ± standard deviation and each value was compared to day 0. *P*-values < 0.05 were considered significant. n = 4. *, $p < 0.05$; **, $p < 0.01$; ***, $p < 0.001$.

2. Supplementary Table

Table S1. Primer details for *EpCAM*, *CK19*, and *SOX9*.

Gene Name	Primer Sequences (5' to 3')
Human	
EpCAM	Forward: GAACAATGATGGGCTTTATG<br>Reverse: TGAGAATTCAGGTGCTTTTT
CK19 (KRT19)	Forward: TCCGAACCAAGTTTGAGACG<br>Reverse: CCCTCAGCGTACTGATTTCC
SOX9	Forward: GAGGAAGTCGGTGAAGAACG<br>Reverse: ATCGAAGGTCTCGATGTTGG

© 2020 by the authors. Licensee MDPI, Basel, Switzerland. This article is an open access article distributed under the terms and conditions of the Creative Commons Attribution (CC BY) license (http://creativecommons.org/licenses/by/4.0/).
